# Development of antiviral LNA gapmers targeting functional stem-loop structures in tick-borne flavivirus genomes

**DOI:** 10.1016/j.omtn.2026.103144

**Published:** 2026-10-15

**Authors:** Shunsuke Okamoto, Mizuki Fukuta, Yuji Fujii, Minato Hirano, Yusuke Echigoya, Kentaro Yoshii

**Affiliations:** 1 Department of Viral Ecology, National Research Center for the Control and Prevention of Infectious Diseases, Nagasaki University, Nagasaki 852-8523, Japan; 2 Nihon University Veterinary Research Center, Fujisawa, Kanagawa 252-0880, Japan; 3 Laboratory of Biomedical Science, Department of Veterinary Medicine, College of Bioresource Sciences, Nihon University, Fujisawa, Kanagawa 252-0880, Japan

**Keywords:** MT: oligonucleotides: therapies and applications, tick-borne encephalitis virus, antisense oligonucleotide gapmer, locked nucleic acid, RNA secondary structures, antiviral nucleic acid therapeutics

## Abstract

Antisense oligonucleotide gapmers degrade target RNA in a sequence-specific and RNase H-dependent manner and are emerging as a promising antiviral strategy. Locked nucleic acid-modified gapmers have shown activity against several pathogenic viruses. Tick-borne encephalitis virus is widely distributed across Eurasia, with approximately 10,000 cases reported annually, and the absence of approved therapies represents a significant public health concern. In this study, we designed 71 locked nucleic acid-modified gapmers targeting structural elements within the untranslated regions of the single-stranded positive-sense RNA genome of tick-borne encephalitis virus. The antiviral efficacy of gapmers selected via *in silico* analysis was evaluated using a replicon system and infectious virus assays. Gapmers targeting stem-loop structures in the 3′ untranslated region effectively inhibited viral replication. Optimizing the length and composition of the lead gapmers further enhanced antiviral potency. Sequence alignment analysis revealed that the target regions of the optimized gapmers were highly conserved among the three major subtypes of tick-borne encephalitis virus. Accordingly, the optimized gapmers markedly inhibited replication of a highly pathogenic strain, suggesting broad-spectrum activity. Collectively, our findings support the development of locked nucleic acid-modified gapmers as therapeutics against tick-borne encephalitis virus and other tick-borne flaviviruses.

## Graphical abstract

**Figure:**
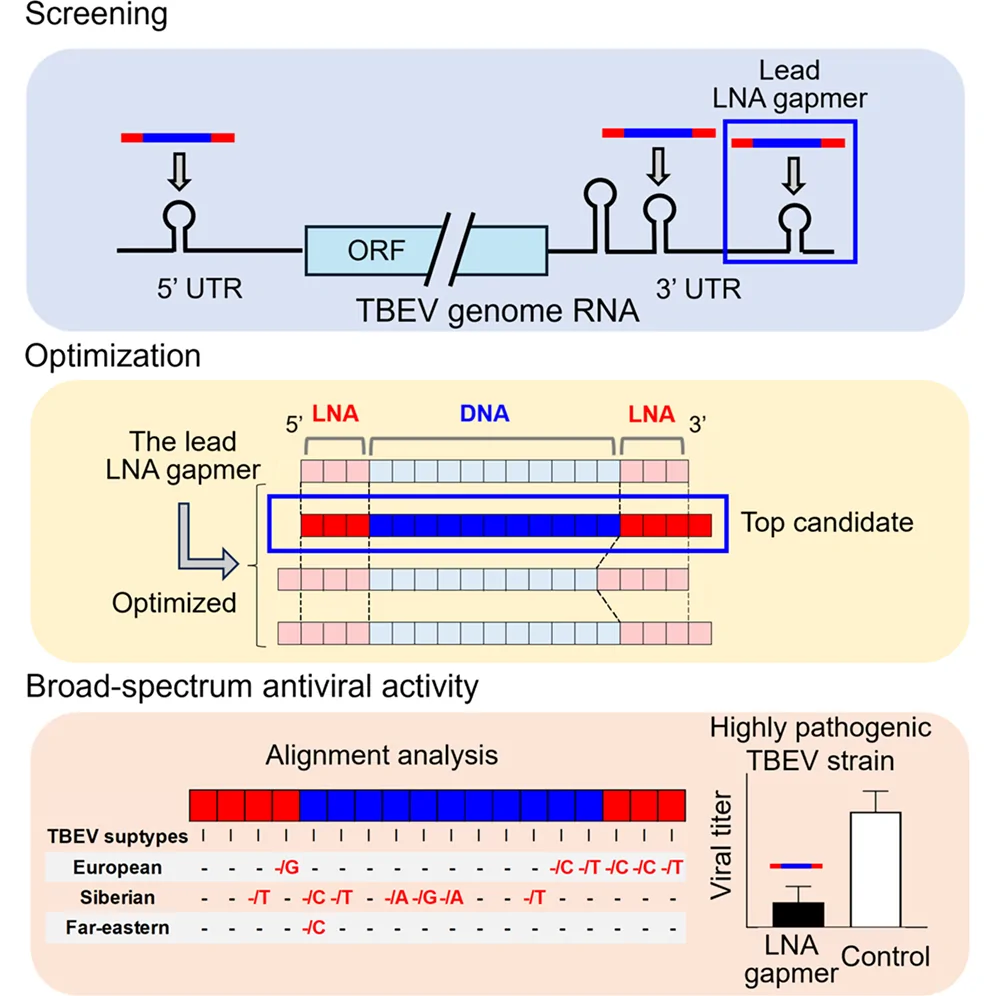

## Teaser

Yoshii and colleagues demonstrated the antiviral efficacy of LNA gapmers targeting conserved 3′ UTR stem-loop structures in tick-borne encephalitis virus (TBEV). Optimization of gapmer length and LNA composition enhanced the antiviral activity supporting conserved viral RNA structures as promising targets for the development of antiviral LNA gapmers.

## Introduction

Oligonucleotide therapeutics are chemically synthesized short nucleic acids that exert pharmacological effects through sequence-specific interactions with target nucleic acids.^1^ Among these, RNA-targeting antisense oligonucleotides (ASOs) have emerged as a promising antiviral.^2^^,^^3^ ASOs are classified into steric-blocking ASOs and gapmers.^4^ Steric-blocking ASOs modulate target RNA function through steric hindrance.^4^ In contrast, gapmers induce target RNA cleavage through RNase H1 activity. These gapmers consist of a central DNA region flanked by modified nucleic acid wings.^5^ Their design requires an appropriate DNA gap length for RNase H1 activity while optimizing oligonucleotide length and modification content for target affinity.^6^^,^^7^ Fomivirsen, an ASO targeting cytomegalovirus (CMV) mRNA, has been approved for CMV retinitis.^1^ Positive-sense RNA viruses use their genomic RNA as mRNA, making the viral genome an attractive target for sequence-specific antiviral ASOs. Furthermore, targeting conserved sequences across viral strains and species may enable broad-spectrum activity, highlighting their adaptability for rapid responses to emerging viral threats.

*Orthoflaviviruses*, including Japanese encephalitis virus (JEV) and tick-borne encephalitis virus (TBEV), are major arthropod-borne pathogens of global concern,^8^ and no specific antiviral treatment has been approved.^9^ TBEV, a representative tick-borne flavivirus, can cause severe encephalitis.^10^ TBEV is divided into at least three subtypes. These viruses are widely distributed across Eurasia, causing more than 10,000 human cases annually.^11^^,^^12^ The European and Siberian subtypes are associated with relatively low mortality rates of approximately 1%–2% and 6%–8%, respectively, whereas the Far Eastern subtype can reach mortality rates of up to 30%.^13^ In recent years, climate change has altered the distribution of the primary vectors, *Ixodes ricinus* and *Ixodes persulcatus*, facilitating the expansion of TBEV-endemic regions.^14^ Our previous studies suggest that TBEV circulates in broader regions not previously recognized as endemic.^15^^,^^16^

TBEV has a positive-sense, single-stranded RNA genome,^17^ comprising a 5′ untranslated region (UTR), a single open reading frame encoding structural and nonstructural proteins, and a 3′ UTR.^18^ The 3′ UTR is divided into a variable region and a core element. This core element is highly conserved among tick-borne flaviviruses and contains multiple stem-loop (SL) structures.^12^ Interactions between these structures and host proteins are essential for viral replication.^19^^,^^20^^,^^21^ Accordingly, SL structures within the core element represent potential targets for ASOs. ASOs targeting functional regions of the 3′ UTR have been reported for mosquito-borne *orthoflaviviruses*.^22^^,^^23^^,^^24^^,^^25^^,^^26^^,^^27^ We previously developed locked nucleic acid (LNA)-modified gapmers targeting 3′ UTR SL structures within JEV genome.^28^ LNA modifications improve ASO target affinity and nuclease resistance.^29^ LNA gapmers have been developed for several pathogenic RNA viruses, including influenza A virus,^30^ severe acute respiratory syndrome coronavirus 2 (SARS-CoV-2),^30^^,^^31^^,^^32^^,^^33^^,^^34^^,^^35^^,^^36^^,^^37^ and hepatitis C virus (HCV).^38^^,^^39^ However, the antiviral activity of LNA gapmers against TBEV has not yet been evaluated.

In this study, we designed 71 LNA gapmers targeting SL structures within the TBEV UTR. Candidates for *in vitro* evaluation were selected based on low predicted off-target effects and high melting temperatures (Tm). The selected LNA gapmers were screened using both a TBEV replicon system and infectious virus assays. Lead gapmers were further optimized in length and composition to enhance antiviral activity. The optimized gapmers were then validated *in vitro* against a highly pathogenic TBEV strain to assess their potency and applicability. These findings establish a framework for the development of TBEV RNA-targeting LNA gapmers.

## Results

### Design and selection of LNA gapmers targeting the UTR of TBEV

Based on the critical role of the 3′ UTR in *orthoflavivirus* replication, we previously demonstrated the antiviral activity of LNA gapmers targeting the 3′ UTR in JEV.^28^ We therefore sought to use LNA gapmers for the specific inhibition of TBEV replication in this study. Fifty-one candidate LNA gapmers were designed by sliding a target window one nucleotide at a time across SL structures within the conserved core element of the 3′ UTR of tick-borne flaviviruses (Figure 1A). The 17-mer length and LNA composition of the gapmers were based on our previous JEV study. Candidates were evaluated based on predicted off-target effects, the presence of toxic sequence motifs, and Tm values. Based on these criteria, nine candidates, designated LNA gapmers 1–9 (ASO IDs 12, 16, 25, 31, 43, 45, 46, 47, and 48 in Table S1), were selected for specific SL structures (Figure 1B; Table 1; Table S1).

**Figure 1:**
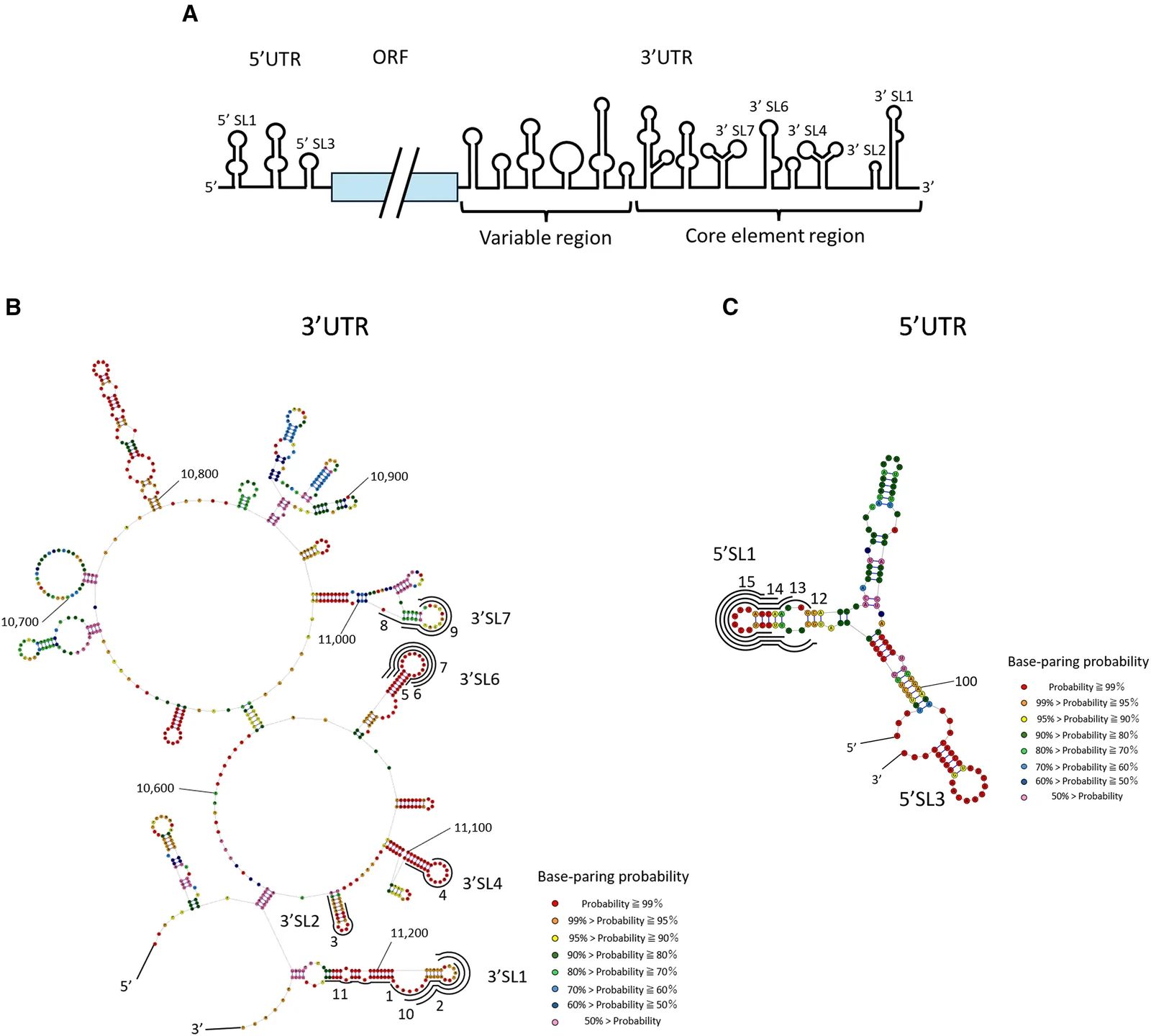
Secondary structures in the 5′ and 3′ UTRs of the TBEV Oshima 5–10 strain (A) Schematic representation of the genome of the TBEV Oshima 5–10 strain (GenBank: AB062063.2). The TBEV genome consists of a 5′ UTR, a single open reading frame (ORF), and a 3′ UTR. The 3′ UTR is divided into two domains: a 5′-terminal variable region and a 3′-terminal core element. The 5′ UTR contains three SL structures, whereas the 3′ UTR contains multiple SL structures. (B and C) LNA gapmers targeting SL structures in the UTRs of the TBEV genomic RNA. RNA secondary structures of the 5′ UTR (1–131 nt) and the 3′ UTR (10,534–11,258 nt) were predicted as minimum free energy structures using the RNAfold program. Targeted SL structures were selected based on low structural complexity in surrounding regions and high conformational probability. Nucleotides are color-coded according to base-pairing probability, as indicated in the figures. LNA gapmer target sites are indicated by black lines and labeled with the corresponding numbers. Smaller font numbers indicate nucleotide positions within the viral genome.

**Table 1:** TBEV-targeting LNA gapmers empirically evaluated in this study

Name	Sequence (5′→3′)	Targeted nucleotides in the TBEV genome (GenBank: AB062063.2)	Tm value (°C)	Length (mer)
LNA gapmer 1	T ˆ ^m^C ˆ ^m^C ˆ t ˆ t ˆ g ˆ t ˆ c ˆ a ˆ g ˆ a ˆ c ˆ t ˆ a ˆ T ˆ ^m^C ˆ T	11,212–11,228	89	17
LNA gapmer 2	T ˆ G ˆ T ˆ c ˆ a ˆ g ˆ a ˆ c ˆ t ˆ a ˆ t ˆ c ˆ t ˆ g ˆ T ˆ G ˆ T	11,208–11,224	69	17
LNA gapmer 3	^m^C ˆ ^m^C ˆ ^m^C ˆ c ˆ c ˆ c ˆ t ˆ c ˆ t ˆ a ˆ t ˆ t ˆ g ˆ a ˆ G ˆ G ˆ G	11,156–11,172	82	17
LNA gapmer 4	A ˆ A ˆ G ˆ a ˆ g ˆ a ˆ g ˆ t ˆ t ˆ g ˆ c ˆ c ˆ a ˆ a ˆ T ˆ T ˆ T	11,108–11,124	71	17
LNA gapmer 5	A ˆ T ˆ G ˆ t ˆ t ˆ c ˆ g ˆ g ˆ c ˆ c ˆ t ˆ t ˆ g ˆ t ˆ ^m^C ˆ A ˆ T	11,053–11,037	72	17
LNA gapmer 6	G ˆ T ˆ T ˆ c ˆ g ˆ g ˆ c ˆ c ˆ t ˆ t ˆ g ˆ t ˆ c ˆ a ˆ T ˆ G ˆ G	11,035–11,051	73	17
LNA gapmer 7	T ˆ T ˆ ^m^C ˆ g ˆ g ˆ c ˆ c ˆ t ˆ t ˆ g ˆ t ˆ c ˆ a ˆ t ˆ G ˆ G ˆ T	11,034–11,050	87	17
LNA gapmer 8	T ˆ ^m^C ˆ G ˆ g ˆ g ˆ a ˆ t ˆ c ˆ g ˆ a ˆ c ˆ c ˆ a ˆ t ˆ T ˆ T ˆ T	10,981–10,997	79	17
LNA gapmer 9	^m^C ˆ G ˆ G ˆ g ˆ a ˆ t ˆ c ˆ g ˆ a ˆ c ˆ c ˆ a ˆ t ˆ t ˆ T ˆ T ˆ ^m^C	10,980–10,996	73	17
LNA gapmer 10	^m^C ˆ ^m^C ˆ G ˆ a ˆ g ˆ t ˆ c ˆ a ˆ c ˆ t ˆ c ˆ a ˆ t ˆ c ˆ A ˆ ^m^C ˆ ^m^C	11,229–11,245	62	17
LNA gapmer 11	T ˆ T ˆ G ˆ t ˆ c ˆ a ˆ g ˆ a ˆ c ˆ t ˆ a ˆ t ˆ c ˆ t ˆ G ˆ T ˆ G	11,210–11,225	83	17
LNA gapmer 12	^m^C ˆ T ˆ G ˆ t ˆ t ˆ g ˆ c ˆ t ˆ a ˆ t ˆ c ˆ c ˆ g ˆ a ˆ A ˆ G ˆ ^m^C	27–43	63	17
LNA gapmer 13	G ˆ T ˆ T ˆ g ˆ c ˆ t ˆ a ˆ t ˆ c ˆ c ˆ g ˆ a ˆ a ˆ g ˆ ^m^C ˆ A ˆ A	25–41	70	17
LNA gapmer 14	T ˆ G ˆ ^m^C ˆ t ˆ a ˆ t ˆ c ˆ c ˆ g ˆ a ˆ a ˆ g ˆ c ˆ a ˆ A ˆ A ˆ ^m^C	23–39	61	17
LNA gapmer 15	^m^C ˆ T ˆ A ˆ t ˆ c ˆ c ˆ g ˆ a ˆ a ˆ g ˆ c ˆ a ˆ a ˆ a ˆ ^m^C ˆ G ˆ ^m^C	21–37	49	17
LNA gapmer 1A	T ˆ ^m^C ˆ ^m^C ˆ t ˆ t ˆ g ˆ t ˆ c ˆ a ˆ g ˆ a ˆ c ˆ t ˆ a ˆ T ˆ ^m^C ˆ T ˆ G	11,212–11,229	84	18
LNA gapmer 1B	^m^C ˆ T ˆ ^m^C ˆ ^m^C ˆ t ˆ t ˆ g ˆ t ˆ c ˆ a ˆ g ˆ a ˆ c ˆ t ˆ A ˆ T ˆ ^m^C ˆ T	11,211–11,228	93	18
LNA gapmer 1C	^m^C ˆ T ˆ ^m^C ˆ ^m^C ˆ t ˆ t ˆ g ˆ t ˆ c ˆ a ˆ g ˆ a ˆ c ˆ t ˆ a ˆ T ˆ ^m^C ˆ T ˆ G	11,211–11,229	84	19
LNA gapmer 6A	G ˆ T ˆ T ˆ C ˆ g ˆ g ˆ c ˆ c ˆ t ˆ t ˆ g ˆ t ˆ c ˆ a ˆ t ˆ G ˆ G ˆ T	11,035–11,052	94	18
LNA gapmer 6B	T ˆ G ˆ T ˆ T ˆ c ˆ g ˆ g ˆ c ˆ c ˆ t ˆ t ˆ g ˆ t ˆ c ˆ A ˆ T ˆ G ˆ G	11,034–11,051	76	18
LNA gapmer 6C	T ˆ G ˆ T ˆ T ˆ c ˆ g ˆ g ˆ c ˆ c ˆ t ˆ t ˆ g ˆ t ˆ c ˆ a ˆ t ˆ G ˆ G ˆ T	11,034–11,052	82	19
Control LNA gapmer 1	A ˆ ^m^C ˆ T ˆ c ˆ t ˆ c ˆ g ˆ t ˆ c ˆ a ˆ a ˆ c ˆ c ˆ A ˆ A ˆ T	NA	71	16
Control LNA gapmer 2	G ˆ T ˆ A ˆ a ˆ c ˆ t ˆ c ˆ g ˆ t ˆ c ˆ g ˆ t ˆ a ˆ A ˆ ^m^C ˆ A	NA	66	16
Control LNA gapmer 3	^m^C ˆ G ˆ A ˆ a ˆ t ˆ a ˆ g ˆ t ˆ t ˆ a ˆ g ˆ t ˆ a ˆ G ˆ ^m^C ˆ G	NA	32	16

N, LNA; n, DNA; m, C5-methylcytosine; ˆ, phosphorothioate; NA, not applicable.

To evaluate the antiviral activity of LNA gapmers, we used a replicon system, a self-replicating RNA construct that does not produce infectious virions in cells.^40^ Their inhibitory effects on viral RNA replication were assessed in baby hamster kidney (BHK-21) cells transfected with a luciferase-expressing TBEV replicon (Figure 2A) and subsequently treated with the respective LNA gapmers. All tested LNA gapmers except LNA gapmer 3 significantly reduced luciferase activity by 64%–84% at 1.0 μM, compared with the untreated control (*p* < 0.001; Figure 2B).

**Figure 2:**
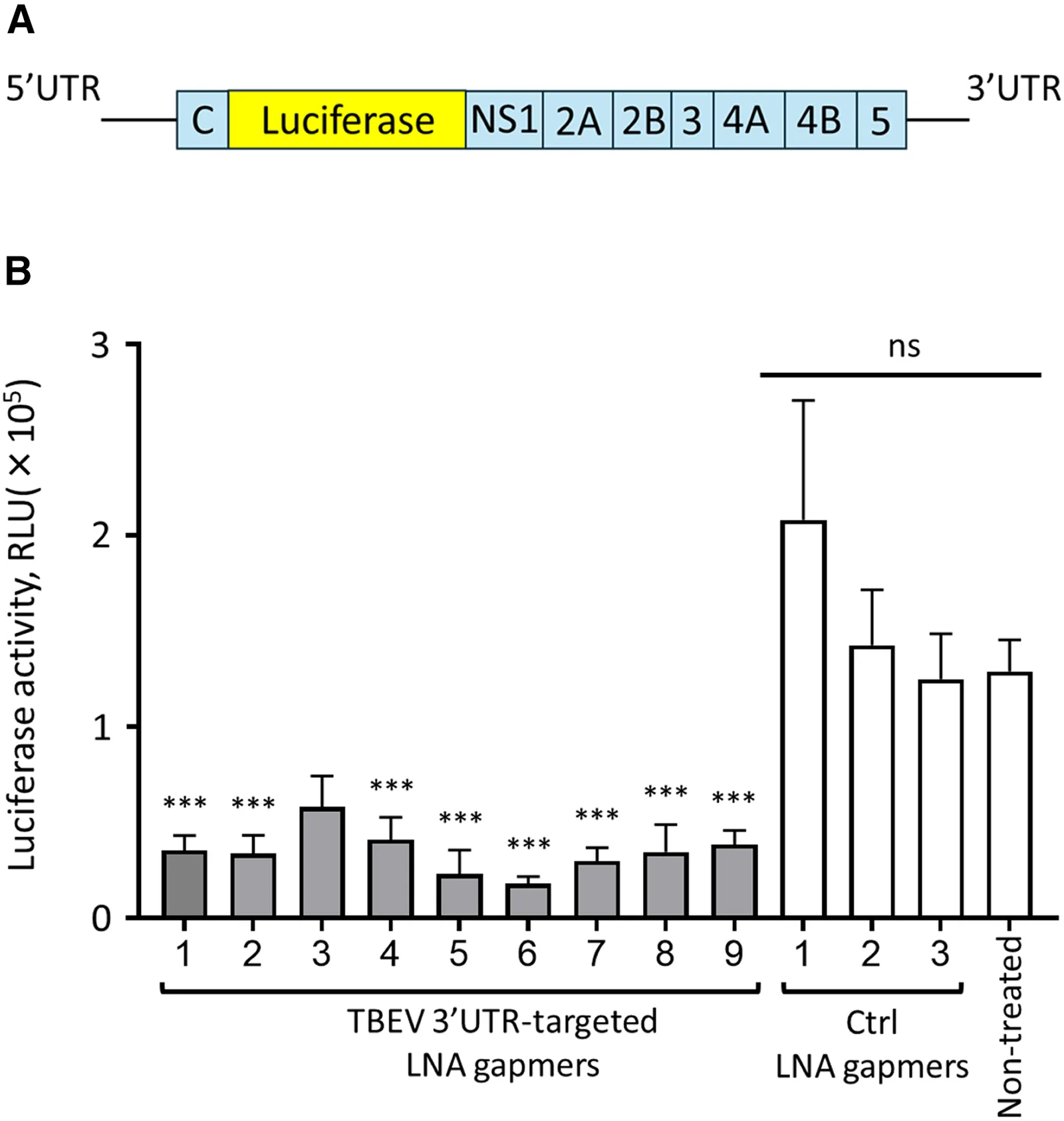
Evaluation of the antiviral activity of LNA gapmers using a TBEV replicon system (A) Schematic representation of the TBEV replicon Oshima luc2A. Oshima luc2A is a replicon derived from the genome of the TBEV Oshima 5–10 strain, in which the coding region for structural proteins is replaced with a luciferase reporter gene. (B) *In vitro* screening of LNA gapmers targeting the 3′ UTR of the TBEV genome, assessed using a luciferase assay. BHK-21 cells were transfected with 100 ng/well of the Oshima luc2A and 1 μM of TBEV 3′ UTR-targeting or control (Ctrl) LNA gapmers using Lipofectamine RNAiMAX. Data are presented as the mean (SD) from four independent experiments. Statistical analysis was performed using one-way ANOVA followed by Tukey-Kramer multiple comparison test: ∗∗∗*p* < 0.001 vs. non-treated; ns, not significant.

We next examined LNA gapmers 1, 2, and 4–9, which were effective in the replicon system, against infectious TBEV. Additionally, to further explore effective target sites within the 3′ UTR, we included LNA gapmer 10 (ASO ID 15), which targets a slightly shifted region within the 3′SL1 compared with LNA gapmers 1 and 2 (Figure 1B). To validate SL structures as effective targets for LNA gapmers, we also designed eight additional candidates (ASO IDs 52–59) and selected one representative, LNA gapmer 11 (ASO ID 58), targeting a double-stranded region.

SL structures within the 5′ UTR have also been reported to be essential for flaviviral replication.^41^^,^^42^ Therefore, we predicted SL structures within the 5′ UTR and explored them as alternative targets for LNA gapmers. Based on their conformational probabilities (Figure 1C), two SLs, 5′SL1 and 5′SL3, were selected for gapmer design. To target these structures, we designed 12 consecutive candidate LNA gapmers (ASO IDs 60–71) at single-nucleotide intervals, comprising 8 and 4 candidates for 5′SL1 and 5′SL3, respectively (Table 1). *In silico* analysis against the human RNA database indicated that the cluster of LNA gapmers targeting 5′SL1 had lower predicted off-target potential than those targeting 5′SL3. Four candidates, LNA gapmers 12–15 (Table 1; Figure 1C), were selected at every other nucleotide position to provide representative coverage of the region.

BHK-21 cells were infected with the TBEV Oshima 5–10 strain at a multiplicity of infection (MOI) of 0.01, followed by transfection with each LNA gapmer at 0.5 μM. Among the tested LNA gapmers, LNA gapmers 1, 6, 8, 9, and 12 significantly inhibited viral replication (Figure 3A). Furthermore, LNA gapmers targeting 3′ UTR SL structures (1, 6, 8, and 9) exhibited greater inhibitory effects than LNA gapmer 12 targeting a 5′ UTR SL (*p* < 0.01). These results support the suitability of the 3′ UTR SL structures as targets for LNA gapmer-mediated inhibition of infectious TBEV replication (Figure 3A). To assess the potential off-target effects in BHK cells, we searched the golden hamster genome for sequence complementary to LNA gapmers 1, 6, 8, and 9 using GGGenome. One perfectly matched sequence was identified for LNA gapmer 1, whereas no perfect matches were found for LNA gapmers 6, 8, or 9 (Table 1). We also assessed the cytotoxicity of LNA gapmers 1 and 6, selected for their greater antiviral efficacy and conservation of target regions. LNA gapmer 1 showed only a modest reduction in cell viability following transfection (Figure 3B). In contrast, LNA gapmer 6 resulted in approximately 60% cell viability at 0.5 μM, suggesting that cytotoxicity may have partly influenced its apparent antiviral effect.

**Figure 3:**
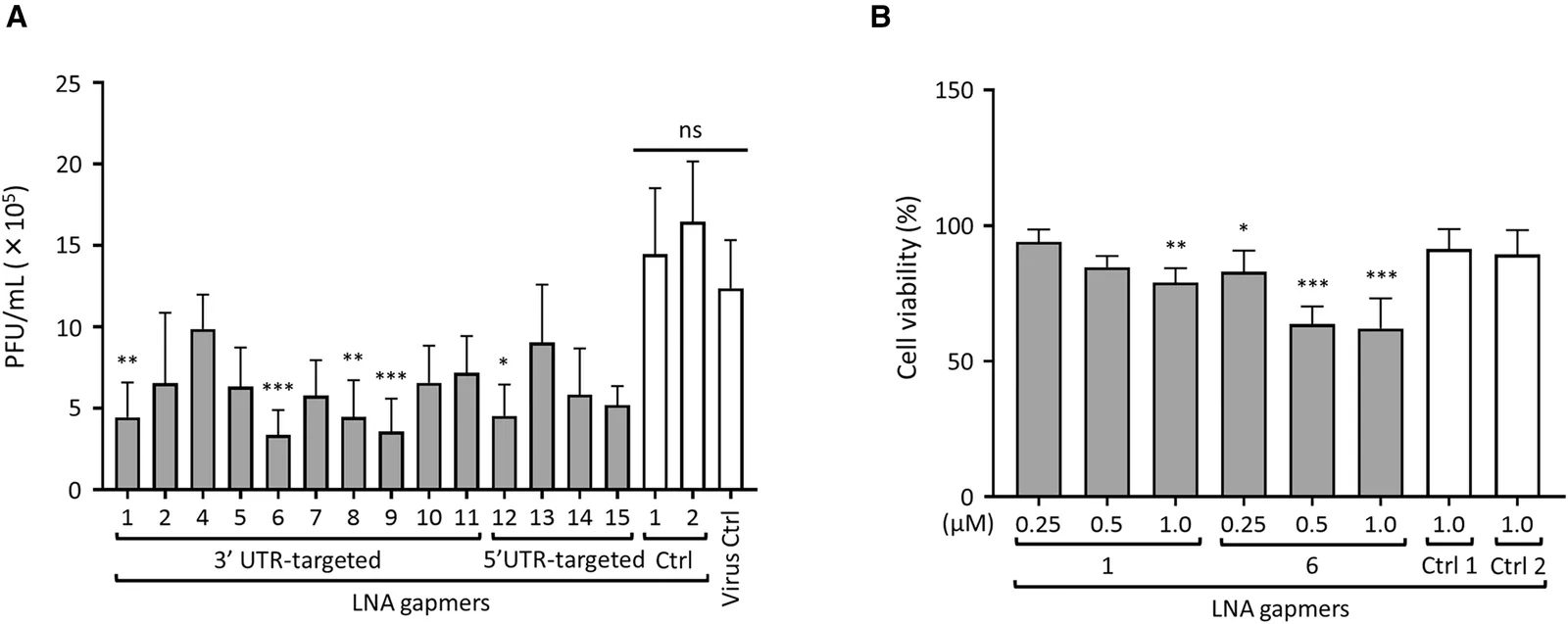
Inhibitory effects of TBEV genome-targeting LNA gapmers on viral replication and cell viability in BHK-21 cells (A) Inhibitory effects of LNA gapmers on TBEV replication, assessed using a plaque assay. BHK-21 cells were infected with the TBEV Oshima 5–10 strain at a MOI of 0.01 and transfected with 0.5 μM TBEV genome-targeting or Ctrl LNA gapmers using Lipofectamine RNAiMAX. Data are presented as the mean (SD) from at least three independent experiments. Statistical analysis was performed using one-way ANOVA followed by Tukey-Kramer multiple comparison test: ∗*p* = 0.011, ∗∗*p* < 0.01, ∗∗∗*p* < 0.001 vs. virus Ctrl; ns, not significant. (B) Cytotoxicity of TBEV genome-targeting and Ctrl LNA gapmers in BHK-21 cells. Cells were transfected with LNA gapmers at concentrations ranging from 0.25 to 1.0 μM. Cell viability was evaluated using the CCK-8 assay, with untreated cells defined as 100% viability. Data are presented as the mean (SD) from three independent experiments. Statistical analysis was performed using the Dunnett’s multiple comparisons test: ∗*p* = 0.043, ∗∗*p* < 0.01, ∗∗∗*p* < 0.001 vs. negative control.

To assess the potential hybridization-dependent off-target effects of LNA gapmers in humans, we searched the human genome and RNA databases for complementary sequences using GGGenome. No perfectly complementary regions were identified in the human genome and pre-spliced RNA databases for LNA gapmers 1, 6, 8, or 9. Potential off-target sequences containing one mismatch or gap in the human genome and pre-spliced RNA databases are shown in Tables S2 and S3, respectively. Furthermore, no complementary regions allowing up to one mismatch or gap were found in the human spliced RNA database (Table 2). This *in silico* analysis suggested that these LNA gapmers have a low predicted risk of off-target effects in human.

**Table 2:** Number of sequences in the human genome and transcriptome complementary to LNA gapmers

	Number of regions complementary to LNA gapmer			
	0 MM or gap	1 MM or gap	2 MMs or gaps	3 MMs or gaps
**Human genome**				

LNA gapmer 1	0	17	1,169	40,366
LNA gapmer 6	0	2	186	6,609
LNA gapmer 8	0	3	73	3,929
LNA gapmer 9	0	1	92	3,312
Control LNA gapmer 1	0	31	1,626	44,191
Control LNA gapmer 2	0	1	315	15,601

**Human pre-spliced RNA**				

LNA gapmer 1	0	26	2,294	search error
LNA gapmer 6	0	3	567	13,131
LNA gapmer 8	0	10	289	search error
LNA gapmer 9	0	1	261	8,337
Control LNA gapmer 1	0	91	4,146	search error
Control LNA gapmer 2	0	10	667	39,965

**Human RNA**				

LNA gapmer 1	0	0	436	15,493
LNA gapmer 6	0	0	170	5,799
LNA gapmer 8	0	0	156	2,849
LNA gapmer 9	0	0	264	3,096
Control LNA gapmer 1	0	0	178	8,291
Control LNA gapmer 2	0	0	59	3,585

Complementary regions were classified according to the total number of mismatches (MMs) or gaps allowed between each LNA gapmer and the complementary sequence. Numbers indicate the number of complementary sequences identified with the indicated number of mismatches or gaps (0–3) between each LNA gapmer and the corresponding sequence. Search error indicates that the number of detected complementary sequences exceeded the displayable limit.

### Optimization of LNA gapmers by adjusting target length and LNA content

To develop more effective LNA gapmers against TBEV, 46 optimized candidates were designed based on LNA gapmers 1 and 6, the top two hits from the initial screening, by adding or shortening single LNA nucleotide at either the 5′ or 3′ end of each LNA wing (Table S4). Among these candidates, those with low predicted off-target effects, high conservation among TBEV strains, and high Tm values were selected as optimized LNA gapmers (Figure 4A). The antiviral activity of the optimized LNA gapmers was then evaluated against TBEV. LNA gapmers 1A and 1B showed significantly stronger inhibitory effects than the original LNA gapmer 1 (Figure 4B). In contrast, LNA gapmers 6A, 6B, and 6C did not exhibit enhanced antiviral efficacy compared with LNA gapmer 6. These findings indicated that the antiviral efficacy of LNA gapmers can be enhanced through optimization of sequence length and LNA modification patterns.

**Figure 4:**
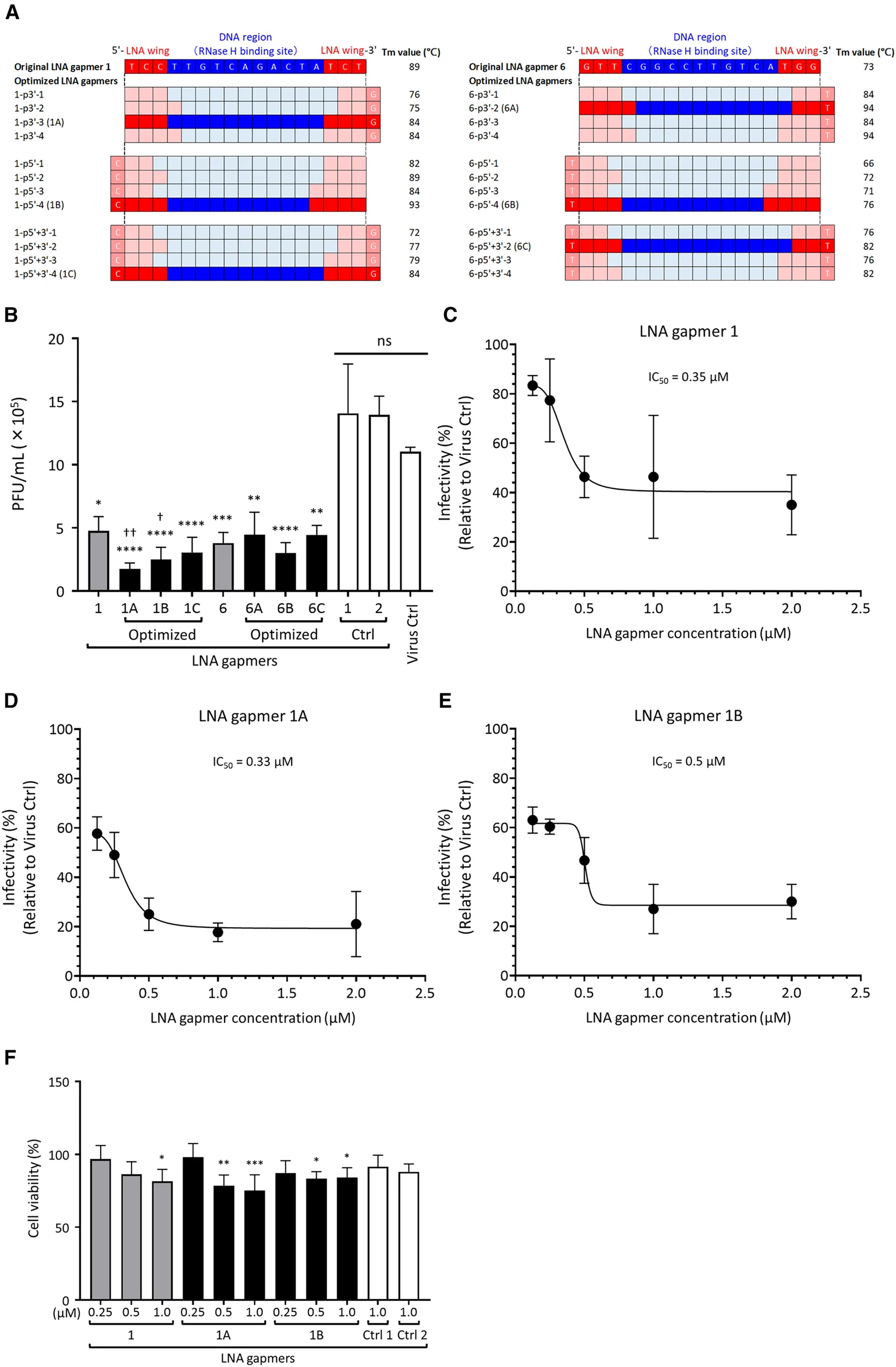
Antiviral activity of optimized LNA gapmers targeting the TBEV genome in BHK-21 cells (A) Design of optimized LNA gapmer candidates. The 5′ and/or 3′ ends of the original LNA gapmers 1 and 6 were modified by adding LNA nucleotides. Similarly, the LNA wings were extended inward at the 5′ and/or 3′ ends. Candidates with high Tms were selected as optimized LNA gapmers. (B) Inhibitory effects of optimized LNA gapmers targeting the 3′ UTR of the TBEV genome, measured using a plaque assay. BHK-21 cells were infected with the TBEV Oshima 5–10 strain at an MOI of 0.01 for 1 h and then transfected with original (1 and 6), optimized (1A, 1B, 1C, 6A, 6B, and 6C), or Ctrl LNA gapmers via lipofection. Data are presented as the mean (SD) of PFU/mL from at least three independent experiments. Statistical analysis was performed using one-way ANOVA followed by the Tukey-Kramer multiple comparison test (∗*p* = 0.014, ∗∗*p* < 0.01, ∗∗∗*p* < 0.001, ∗∗∗∗*p* < 0.0001 vs. virus Ctrl) and Dunnett’s test (^†^*p* = 0.034, ^††^*p* < 0.01 vs. original LNA gapmer). (C–E) Dose-dependent antiviral effects of the original (1) and optimized (1A and 1B) LNA gapmers, measured using a plaque assay. BHK-21 cells infected with TBEV at an MOI of 0.01 were transfected with LNA gapmers 1, 1A, or 1B at the indicated concentrations. Data are presented as the mean (SD) from three independent experiments. IC50 values represent the concentration required to reduce viral titers by 50%. (F) Cytotoxicity of LNA gapmers in BHK-21 cells. Cells were transfected with original (1), optimized (1A and 1B), or Ctrl LNA gapmers at concentrations ranging from 0.25 to 1.0 μM. Cell viability was assessed using the CCK-8 assay, with untreated cells defined as 100% viability. Data are presented as the mean (SD) from four independent experiments. Statistical analysis was performed using the Dunnett’s multiple comparison test: ∗*p* = 0.016 for LNA gapmer 1 (1.0 μM); *p* = 0.034, and 0.048 for LNA gapmer 1B (0.5 and 1.0 μM), respectively; ∗∗*p* < 0.01, all vs. negative control.

Next, to compare the dose-dependent efficacy of the LNA gapmers, we determined their IC_50_ values and assessed cytotoxicity for LNA gapmers 1, 1A, and 1B. The IC_50_ values were 0.35, 0.33, and 0.5 μM for LNA gapmers 1, 1A, and 1B, respectively (Figures 4C–4E). Among these, LNA gapmer 1A exhibited the lowest IC_50_. Although cell viability was significantly reduced, the effect was modest across all optimized LNA gapmers tested. As shown in Figure 4F, LNA gapmer 1A caused the smallest reduction in cell viability. Based on its potency and cytotoxicity profile, the LNA gapmer 1A was selected as the lead antiviral candidate against TBEV.

### Evaluation of broad-spectrum antiviral activity and sequence specificity of LNA gapmers

To evaluate the potential broad-spectrum antiviral activity of the optimized LNA gapmers across TBEV strains, we first examined sequence conservation of their target sites using 151 TBEV genome sequences from the National Center for Biotechnology Information (NCBI) database. The target regions of the lead candidates, LNA gapmers 1A and 1B, showed 82%, 84%, and 96% sequence conservation among the European, Siberian, and Far Eastern subtypes, respectively, based on 61 representative TBEV strains covering the three major subtypes (Figure 5A).

**Figure 5:**
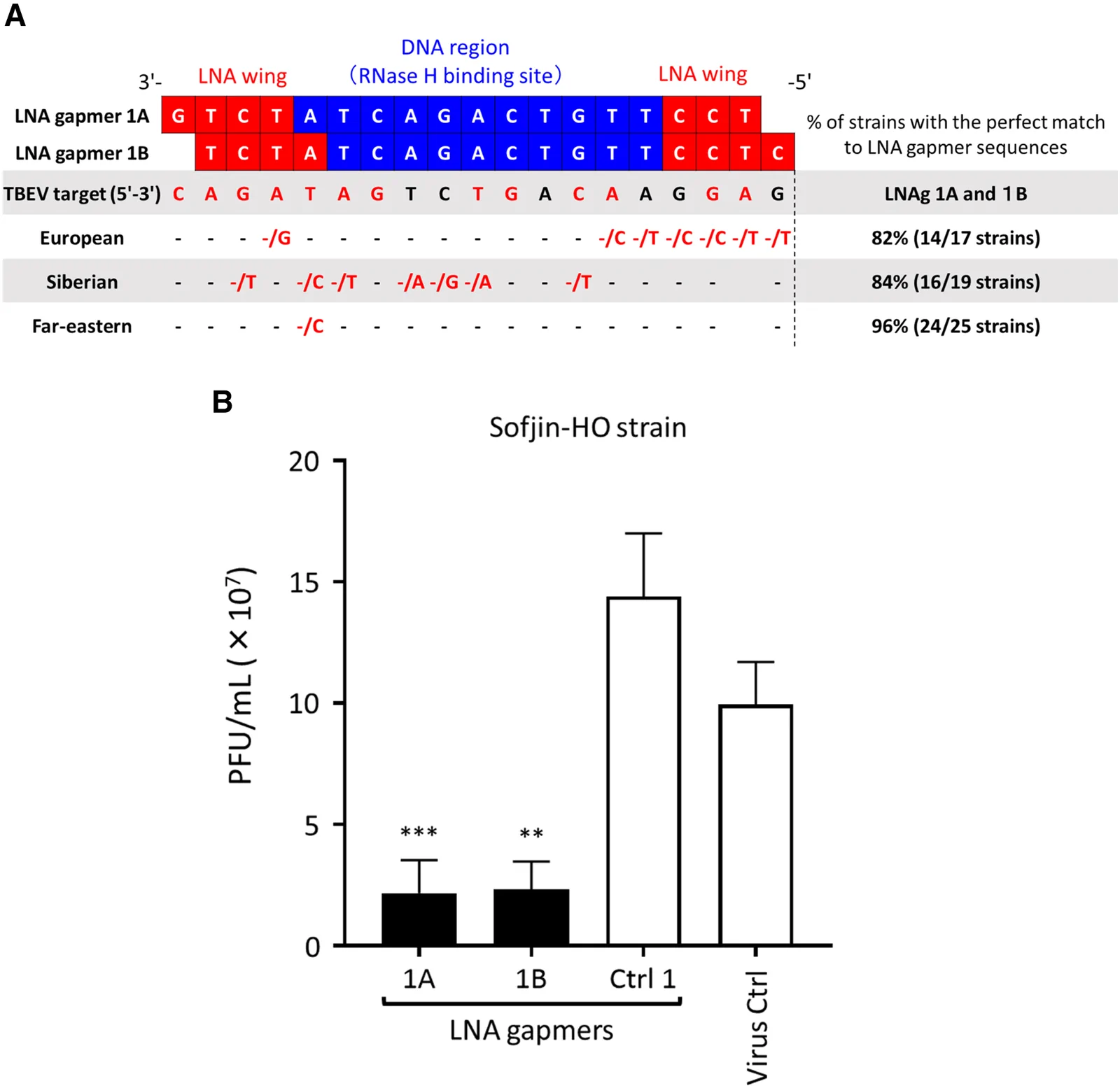
Inhibitory effects of LNA gapmers on replication of a Far Eastern subtype strain of TBEV in BHK-21 cells (A) Sequence conservation analysis of TBEV target regions for LNA gapmers 1A and 1B. Genomic RNA sequences from 61 TBEV strains representing three subtypes were retrieved from the NCBI GenBank database and analyzed for conservation within the target regions of LNA gapmers 1A and 1B. The “TBEV target” corresponds to nucleotides 11,211–11,229 of the TBEV Oshima 5–10 genome. Dashes indicate nucleotides identical to those in the TBEV target. Nucleotides following a slash indicate substitutions observed among TBEV strains. (B) Antiviral efficacy of LNA gapmers (0.5 μM) against the TBEV Sofjin-HO strain (MOI 0.01) in BHK-21 cells. Viral titers (PFU/mL) were measured using a plaque assay. Data are presented as the mean (SD) from four independent experiments. Statistical analysis was performed using one-way ANOVA followed by the Tukey-Kramer multiple comparison test: ∗∗*p* < 0.01, ∗∗∗*p* < 0.001 vs. virus Ctrl.

Next, we examined the antiviral efficacy of LNA gapmers 1A and 1B against the highly pathogenic TBEV Sofjin-HO strain, which belongs to the Far Eastern subtype and shares an identical target sequence with the Oshima 5–10 strain. We also tested their activity against Langat virus (LGTV), another member of the TBEV serocomplex. LNA gapmers 1A and 1B significantly inhibited replication of the Sofjin-HO strain (Figure 5B), whereas no inhibitory effect was observed against LGTV (Figures S1A and S1B). These findings suggested that the optimized LNA gapmers may exhibit broad-spectrum antiviral activity across TBEV strains while maintaining sequence specificity, as even a single mismatch can impair antiviral efficacy within the TBEV serocomplex.

## Discussion

In this study, we first demonstrated the antiviral efficacy of LNA gapmers against TBEV, a medically important tick-borne flavivirus, *in vitro*. We identified SL structures within the 3′ UTR of the TBEV genomic RNA as suitable targets for LNA gapmer development. In contrast, LNA gapmers targeting a double-stranded region within the 3′ UTR or an SL structure within the 5′ UTR did not efficiently inhibit TBEV replication. Based on these findings, we further optimized active LNA gapmers by adjusting oligonucleotide length and LNA composition, resulting in a significant enhancement of antiviral activity. Notably, the target regions of the optimized LNA gapmers showed >82% sequence conservation among the three major TBEV subtypes, and these gapmers successfully inhibited replication of a highly pathogenic TBEV strain with a conserved target sequence.

Several LNA gapmers targeting SL structures suppressed replication of both TBEV replicons and infectious virus (Figures 1, 2, and 3). Antiviral LNA gapmers directed against SL structures have also been reported for other RNA viruses, including JEV,^28^ influenza A virus,^30^ and SARS-CoV-2.^30^^,^^34^^,^^35^ As previously discussed, interactions between viral SL structures and host proteins are critical for viral replication.^19^^,^^20^^,^^21^ Therefore, disruption of these interactions by LNA gapmers may contribute to antiviral efficacy. Moreover, SL structures tend to be highly conserved among viral species,^43^ supporting their suitability as promising targets for antiviral LNA gapmer development.

Consistent with a previous study,^30^ the present work suggests that effective antiviral LNA gapmers preferentially target loop regions or the 3′ side of SL structures. It has been reported that LNA gapmers efficiently induce degradation of cellular mRNA, and their efficacy is thought to depend in part on the accessibility of RNA structures complementary to the central DNA gap region.^44^ Although the precise mechanism underlying the preferential efficacy of LNA gapmers targeting the 3′ side of SL structures remains unclear, it may relate to the resulting RNA-DNA helical conformation. LNA gapmers targeting the 3′ side of an SL may bind the RNA helix from the major groove,^44^ which is relatively narrow, whereas the opposite side presents a wider minor groove that may be more accessible to RNase H.^45^ Consequently, targeting the 3′ side of SL structures may provide a structural context more favorable for RNase H-mediated cleavage of target RNA.

In the present study, LNA gapmers targeting stem regions within the 3′ UTR and SL structures within the 5′ UTR did not show strong antiviral activity (Figure 3A). The precise reason for the differences in antiviral efficacy between 5′ UTR- and 3′ UTR-targeting LNA gapmers remains unclear. Recent structural studies using NMR and SHAPE-seq have revealed that higher-order RNA conformations within flaviviral UTRs contribute to viral replication and resistance to host exonucleases.^46^^,^^47^ Therefore, differences in RNA conformational features not fully captured by secondary structure predictions may underlie the distinct responses of 5′ UTR- and 3′ UTR-targeting LNA gapmers observed in this study. Although our results support SL structures within the 3′ UTR as promising target regions, screening of other conserved regions of the viral genome, including coding regions, may identify more effective LNA gapmer candidates.

Among the LNA gapmers that were effective against the TBEV replicon, LNA gapmers 1, 6, 8, and 9 inhibited replication of infectious viral particles (Figure 3). Previous studies have similarly reported that steric-blocking LNA ASOs effective against HCV replicons also suppress replication of infectious HCV.^48^ Together, these findings support the utility of replicon systems as simple and rapid screening tools for LNA gapmers against highly pathogenic viruses such as TBEV, for which experiments involving infectious virus are restricted to high-containment facilities.

We further optimized the lead LNA gapmers by adjusting their length and LNA composition to enhance antiviral efficacy (Figure 4A). Although reports on systematic optimization of antiviral LNA gapmers remain limited, we previously demonstrated that altering a single LNA position significantly increased antiviral activity against JEV.^28^ Based on this observation, we hypothesized that extending LNA gapmers by one nucleotide would increase binding affinity to the target RNA and thereby improve antiviral efficacy. Although increasing ASO length can reduce the number of partially complementary sequences in human transcripts,^49^ 17–18 nucleotides have been reported as a reasonable starting point for LNA gapmer design.^7^ In our previous study, 17-mer LNA gapmers also inhibited JEV replication, with no fully complementary sequences identified in the human genome database.^28^ Based on these considerations, we selected 17-mer LNA gapmers for the initial screening, and subsequently extended active candidates to 18–19 nucleotides during optimization. Based on these considerations and previous studies on LNA gapmer design,^28^^,^^36^^,^^50^^,^^51^ we designed multiple optimized LNA gapmers with low predicted off-target effects and high Tm values, and evaluated their inhibitory effects against TBEV (Figure 4; Table S4). This optimization strategy resulted in enhanced antiviral activity, consistent with recent findings reported for anti-SARS-CoV-2 LNA gapmers.^36^ These results suggest that rational adjustment of LNA gapmer length and LNA placement is an effective strategy for improving antiviral activity. However, increasing LNA content while shortening the DNA core may enhance binding stability, potentially improving on-target activity but also increasing the risk of off-target binding.^52^ Therefore, optimization of LNA gapmer design requires balancing antiviral activity, target specificity, and off-target effects. Further screening may identify LNA gapmer candidates with greater antiviral potency at lower concentrations and reduced cytotoxicity.

Sequence alignment analysis revealed high conservation of the LNA gapmer target regions among TBEV subtypes, and the optimized gapmers inhibited the replication of the Sofjin-HO strain (Figure 5), suggesting potential broad-spectrum antiviral activity within TBEV strains. This observation is consistent with our previous findings using anti-JEV LNA gapmers.^28^ However, LNA gapmers targeting TBEV did not suppress LGTV replication, despite the presence of only a single nucleotide mismatch in the target sequence (Figure S1A). As shown in Figure 1 and Figure S1, the LNA gapmer 1A target regions exhibited distinct secondary structures in TBEV and LGTV. In LGTV, the single-stranded region within the target site is shorter than that in TBEV. These observations suggest that differences in RNA secondary structure, combined with sequence mismatches, may attenuate antiviral efficacy. Therefore, both sequence conservation and RNA secondary structure should be considered in the rational design of antiviral LNA gapmers. In this regard, the use of combinations of LNA gapmers targeting distinct RNA structural elements may represent a promising strategy to achieve broader antiviral activity, as demonstrated by steric-blocking ASOs such as AVI-6002 and AVI-6003 developed against Ebola and Marburg viruses.^53^

In conclusion, this study provides the first demonstration of the antiviral activity of LNA gapmers against TBEV replication, supporting further *in vivo* evaluation. Strategies such as the combined use of multiple LNA gapmers or additional modifications, including ligand-conjugated heteroduplex forms, may improve delivery to the central nervous system and help overcome current limitations in antiviral spectrum and *in vivo* efficacy.^53^^,^^54^ Collectively, our findings contribute to the development of ASO-based therapeutics against TBEV and other tick-borne flaviviruses.

## Materials and methods

### Cell and viruses

BHK-21 cells were maintained in Eagle’s minimum essential medium (EMEM; Fujifilm, Tokyo, Japan) supplemented with 8% heat-inactivated fetal bovine serum (FBS) at 37°C in a humidified atmosphere containing 5% CO_2_. Recombinant viruses derived from infectious clones of the TBEV strains Oshima 5–10 and Sofjin-HO were generated as previously described.^55^^,^^56^ The LGTV strain TP-21, a member of the tick-borne flaviviruses, was propagated in BHK-21 cells. Viral titers were determined by plaque assay. Virus stocks were aliquoted and stored at −80°C until use. All experiments involving TBEV were conducted in a biosafety level 3 (BSL-3) laboratory in accordance with the biosafety regulations of Nagasaki University.

### Viral RNA secondary structure prediction

Complete genome sequences of the TBEV strains Oshima 5–10 and Sofjin-HO, and the LGTV TP-21 strain, were obtained from the NCBI GenBank database (GenBank: AB062063.2, AB062064.1, and EU790644.1, respectively). Secondary structures of the 5′ and 3′ UTRs of TBEV, and the 3′ UTR of LGTV were predicted as minimum free energy structures using RNAfold implemented in the ViennaRNA Package (v.2.6.3; http://rna.tbi.univie.ac.at/cgi-bin/RNAWebSuite/RNAfold.cgi; accessed on July 23, 2026) with default parameters.^57^ The predicted secondary structures and corresponding base-pairing probability information were obtained from RNAfold and imported into StructureEditor (v.1.0), provided as part of the RNAstructure software package. The predicted structures were manually rearranged for visualization without altering the predicted base-pairing, and nucleotides were colored according to the base-pairing probabilities calculated by RNAfold.

### LNA gapmer design and synthesis

Seventy-one LNA gapmer sequences, each assigned a unique ASO ID, were designed by sliding a target window one nucleotide at a time across predicted secondary structures in the 5′ and 3′ UTRs of the Oshima 5–10 and Sofjin-HO strains (Table S1). LNA gapmers were designed with a 17-mer LNA-DNA-LNA (3-11-3) configuration based on our previous study, which demonstrated antiviral activity with moderate cytotoxicity.^28^ For the primary design, the highly conserved 3′ UTR core element shared among TBEV strains was selected (Figure 1A). SL structures within the 5′ and 3′ UTRs were identified as target sites based on low structural complexity in surrounding regions and high predicted conformational stability (Figures 1B and 1C).

Potential off-target binding of the designed LNA gapmer sequences to human RNAs was predicted using GGGenome (https://gggenome.dbcls.jp/en/; accessed on November 24, 2024) for initial *in silico* screening. Predicted Tm of the LNA-modified sequences were calculated using the QIAGEN Tm Prediction Tool (https://geneglobe.qiagen.com/us/tools/tm-prediction; accessed November 24, 2024) as an indicator of hybridization stability. Based on previous studies,^28^^,^^49^^,^^58^^,^^59^ candidate sequences were considered to have potential off-target effects if they showed predicted complementarity to human RNAs allowing up to one mismatch or gap, or if they contained four or more consecutive guanines (ASO IDs 26–29). Such candidates were excluded from further selection.

Accordingly, candidates targeting the 3′ UTR for *in vitro* testing were selected based on low predicted off-target effects, absence of consecutive guanine motifs, and high predicted Tm values for each SL structure. The selected sequences were synthesized as LNA gapmers 1–11, as shown in Table 1. For the 5′ UTR, LNA gapmers 12–15 targeting 5′SL1 were selected at every other nucleotide position from candidates with low predicted off-target effects to ensure representative coverage of this region.

To enhance antiviral efficacy, lead LNA gapmers were further optimized based on their *in vitro* activity and/or conservation of target sequences among TBEV strains. The lengths and LNA/DNA compositions of these lead gapmers were optimized to reduce predicted off-target binding to human RNAs and increase predicted Tm values. Control LNA gapmers were obtained from a previous study.^28^ All LNA gapmers were synthesized and HPLC-purified by Ajinomoto Bio-Pharma Services GeneDesign, Inc. (Osaka, Japan).

### *In vitro* screening of antiviral LNA gapmers using a replicon system

The Oshima-REP luc2A plasmid was used to generate luciferase-expressing replicon RNA derived from the Oshima strain, as previously described.^60^ BHK-21 cells were seeded at a density of 2.0 × 10^5^ cells per well in 24-well plates and incubated at 37°C in 5% CO_2_ for 24 h to reach approximately 95% confluency. Cells were transfected with 100 ng/well of Oshima REP luc2A mRNA for 6 h using Lipofectamine MessengerMAX (Thermo Fisher Scientific, Waltham, MA).

LNA gapmer transfection was performed using Lipofectamine RNAiMAX (Thermo Fisher Scientific) as previously described.^28^ Briefly, during replicon transfection, an 80 μL mixture containing LNA gapmers (final concentration: 3.0 μM) and 3 μL of Lipofectamine RNAiMAX was prepared in Opti-MEM (Thermo Fisher Scientific). To obtain a final concentration of 1.0 μM, the LNA mixture was diluted 3-fold with EMEM containing 5% FBS. After removal of the replicon transfection mixture, cells were transfected with 230 μL of the LNA mixture and incubated for 4 h. Following transfection, cells were washed once with EMEM supplemented with 5% FBS and maintained in medium containing 2% FBS. Cells were harvested 48 h after replicon transfection for luciferase assay. Luciferase activity was measured using the Luciferase Assay System (Promega, Madison, WI) and expressed as raw light unit (RLU).

### Validation of antiviral activity using infectious TBEV

BHK-21 cells were seeded at a density of 2.0 × 10^5^ cells per well in 24-well plates and incubated at 37°C in 5% CO_2_. Cells were infected with TBEV at an MOI of 0.01 in EMEM supplemented with 2% FBS for 1 h, with tilting every 15 min. LNA gapmer transfection was performed as described previously. LNA mixtures were diluted to a final concentration of 0.5 μM in EMEM containing 5% FBS. Supernatants were collected 24 h post-infection (hpi) and subjected to plaque assays to determine viral titers. For plaque assays, BHK-21 cells were seeded in 12-well plates. Cells were inoculated with 100 μL of serial 10-fold dilutions of culture supernatants and incubated at 37°C for 1 h. After incubation, 1 mL of overlay medium consisting of minimum essential medium (MEM) containing 1.5% carboxymethyl cellulose (CMC) was added to each well. Cells were further incubated for 3–4 days at 37°C. Monolayers were then fixed with 3.7% formaldehyde in PBS and stained with 0.1% crystal violet. Plaques were counted, and viral titers were expressed as plaque-forming units per milliliter (PFU/mL).

### Cytotoxicity assay

BHK-21 cells were seeded at a density of 0.34 × 10^5^ cells per well in 96-well plates using EMEM supplemented with 8% FBS and incubated for 24 h at 37°C. For lipofection, a 240-μL transfection mixture containing LNA gapmers at final concentrations ranging from 0.25 to 1.0 μM and 3 μL of Lipofectamine RNAiMAX was prepared as described above. Cells were transfected with 40 μL of the transfection mixture and incubated for 4 h. After transfection, cells were washed once with EMEM containing 5% FBS and incubated in 100 μL EMEM supplemented with 2% FBS for 18 h. Cytotoxicity was then assessed using the Cell Counting Kit-8 (CCK-8; Dojindo, Kumamoto, Japan) according to the manufacturer’s instructions. Briefly, 10 μL of CCK-8 solution was added to each well and the plates were incubated for 2 h at 37°C. Absorbance was measured at 450 nm using a SpectraMax iD3 or iD5 microplate reader (Molecular Devices, San Jose, CA).

### Analysis of potential off-target binding of TBEV RNA-targeted LNA gapmers to human RNA and human/hamster genomes

Potential off-target binding sites of LNA gapmers 1, 6, 8, and 9, as well as control LNA gapmers 1 and 2, were analyzed by searching for complementary regions against human RNA sequences (RefSeq human RNA release 220 [September 2023], RefSeq human RNA release 225 [July 2024], and human pre-spliced RNA, RefSeq curated on GRCh38/hg38.p14, D3G 26.03 [March 2026]), human genome (GRCh38/hg38 [December 2013]) using GGGenome. Complementary sequences containing 0–3 mismatches or gaps were identified and classified according to the number of mismatches or gaps (Table 2). The golden hamster genome (MesAur1.0 [March 2013]) was also analyzed using the same methods.

### Conservation analysis of LNA gapmer target regions in TBEV subtypes

A total of 151 TBEV RNA genome sequences corresponding to the target regions of LNA gapmers 1A and 1B were retrieved from the NCBI GenBank database. These sequences included 96 European, 30 Siberian, and 25 Far Eastern subtype strains.

### Statistical analysis

Data are presented as the mean (standard deviation: SD) from at least three independent experiments. For statistical analysis, viral titers were log-transformed and analyzed using one-way analysis of variance (ANOVA), followed by Tukey-Kramer multiple comparison test using GraphPad Prism 8 (GraphPad Software, La Jolla, CA). For the cytotoxicity assay, the Dunnett’s multiple comparison test was performed using GraphPad Prism 8. All statistical tests were two-sided, and *p* < 0.05 was considered statistically significant.

## Data and code availability

The datasets generated and/or analyzed during the current study are available from the corresponding author on reasonable request.

## Acknowledgments

We would like to thank Editage (www.editage.com) for English language editing. This work was supported by JSPS KAKENHI grant (21KK0123, 23K27079, 24K23165, and 25K18370), a grant for research on Japan Program for Infectious Diseases Research and Infrastructure from AMED (JP25fm0208101 and JP25wm0325059), and the cooperative research project program of the National Research Center for the Control and Prevention of Infectious Diseases, Nagasaki University.

## Author contributions

Conceptualization, K.Y.; methodology, S.O., M.F., M.H., Y.E., and K.Y.; resources, M.F., Y.F., and M.H.; investigation, S.O.; formal analysis, S.O.; writing – original draft, S.O.; writing – review and editing, S.O., Y.E., and K.Y.; validation, S.O.; visualization, S.O.; supervision, Y.E. and K.Y.; project administration, K.Y.; funding acquisition, S.O., Y.E., and K.Y.

## Declaration of interests

The authors declare no competing interests.

## Supplemental information

Document S1. Figure S1 and Tables S1–S4

Document S2. Article plus supplemental information
