# Supplementary material for: Development of antiviral LNA gapmers targeting functional stem-loop structures in tick-borne flavivirus genomes
Source: Mol Ther Nucleic Acids. 2026 Oct 15;37(4):103144. doi: 10.1016/j.omtn.2026.103144 (PMC13757288; doi:10.1016/j.omtn.2026.103144)
Supplement: Supplementary file 1 [file mmc1.pdf]

OMTN, Volume *37*

Supplemental information

Development of antiviral LNA gapmers targeting functional stem-loop structures in tick-borne flavivirus genomes

Shunsuke Okamoto, Mizuki Fukuta, Yuji Fujii, Minato Hirano, Yusuke Echigoya, and Kentaro Yoshii

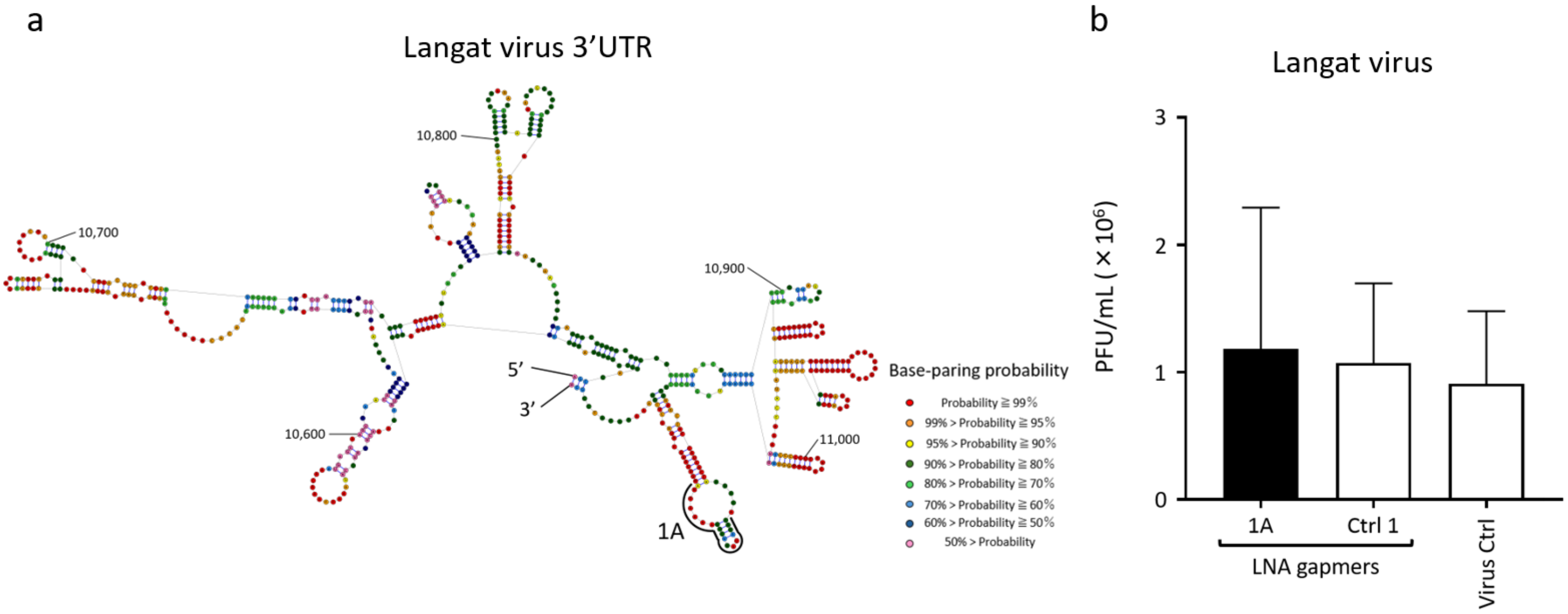

Figure S1: Antiviral activity of LNA gapmer 1A against LGTV.

(a) Predicted RNA secondary structure of the 3’ UTR (10,524-11,099 nt) of the LGTV TP-21 strain (GenBank: EU790644.1), generated as a minimum free energy structure using RNAfold program. The target site for LNA gapmer 1A is indicated by a black line. Smaller font numbers indicate nucleotide positions in the viral genome. Nucleotides are color-coded according to base-pairing probability, as indicated in the figure.

(b) Evaluation of the antiviral activity of LNA gapmer 1A against LGTV.

BHK-21 cells were infected with the LGTV TP-21 strain at a MOI of 0.01 and transfected with 0.5 μM LNA gapmer 1A using Lipofectamine RNAiMAX. Data are presented as mean (SD) from three independent experiments.

Table S1: Design of LNA gapmer candidates.

The selected LNA gapmers are highlighted in blue. The numbers in parentheses indicate the LNA gapmer numbers.

MM, mismatch.

ASO ID	ASO sequence[5'→3'] (17-mer; LNA–DNA–LNA, 3–11–3)	Target region	Target secondary structure	No. of complementary sequences in Human RNA (RefSeq human RNA release 220 (Sep, 2023))				No. of complementary sequences in Hamster genome (Golden hamster genome, MesAur1.0 (Mar, 2013))				GC(%)	Tm value(℃)	No. of TBEV strains with the perfect match to ASO sequences			
				0 MM or gap	1 MM or gap	2 MM or gap	3 MM or gap	0 MM or gap	1 MM or gap	2 MM or gap	3 MM or gap			European subtype	Siberian subtype	Far-eastern subtype	Total
1	CACACATCACCTCCTTG	3'UTR	3'SL1	0	35	1328	21675	0	43	1742	34253	52.9	71	13/16	16/19	8/16	37/51
2	ACACATCACCTCCTTGT	3'UTR	3'SL1	0	16	965	16484	1	22	1320	28673	47.1	78	13/16	16/19	9/17	38/52
3	CACATCACCTCCTTGTC	3'UTR	3'SL1	4	7	874	20207	0	31	1343	29491	52.9	84	13/16	16/19	9/17	38/52
4	ACATCACCTCCTTGTCA	3'UTR	3'SL1	0	8	913	17188	0	24	1054	25322	47.1	80	13/16	16/19	16/25	45/60
5	CATCACCTCCTTGTCAG	3'UTR	3'SL1	0	12	1488	22239	0	22	1104	27488	52.9	67	13/16	17/19	24/25	54/60
6	ATCACCTCCTTGTCAGA	3'UTR	3'SL1	0	6	804	15468	0	13	848	26828	47.1	89	13/16	17/19	24/25	54/60
7	TCACCTCCTTGTCAGAC	3'UTR	3'SL1	0	6	692	13649	0	10	843	22161	52.9	85	13/16	17/19	25/25	55/60
8	CACCTCCTTGTCAGACT	3'UTR	3'SL1	0	6	650	16730	0	18	1019	24167	52.9	81	14/17	17/19	25/25	56/61
9	ACCTCCTTGTCAGACTA	3'UTR	3'SL1	0	6	213	7546	0	14	591	16778	47.1	80	14/17	16/19	24/25	54/61
10	CCTCCTTGTCAGACTAT	3'UTR	3'SL1	0	17	226	9314	0	16	775	19914	47.1	76	14/17	16/19	24/25	54/61
11	CTCCTTGTCAGACTATC	3'UTR	3'SL1	0	17	345	11709	0	21	791	20782	47.1	82	14/17	16/19	24/25	54/61
12 (1)	TCCTTGTCAGACTATCT	3'UTR	3'SL1	0	0	436	15493	1	49	1278	32160	41.1	89	14/17	16/19	24/25	54/61
13	CCTTGTCAGACTATCTG	3'UTR	3'SL1	0	4	302	11703	1	14	936	26465	47.1	64	14/17	17/20	24/25	55/62
14	CTTGTCAGACTATCTGT	3'UTR	3'SL1	0	6	277	12840	2	31	1070	30349	41.1	69	14/17	17/20	24/25	55/62
15 (10)	TTGTCAGACTATCTGTG	3'UTR	3'SL1	0	0	340	12518	2	36	1220	32392	41.1	62	14/17	17/20	24/25	55/62
16 (2)	TGTCAGACTATCTGTGT	3'UTR	3'SL1	0	0	309	14475	0	31	1554	42736	41.1	69	15/17	17/20	23/25	55/62
17	GTCAGACTATCTGTGTC	3'UTR	3'SL1	0	3	256	9965	0	11	849	23948	47.1	93	18/19	17/20	23/25	58/64
18	TCAGACTATCTGTGTCT	3'UTR	3'SL1	0	4	635	16679	0	27	1769	46019	41.1	80	18/19	18/20	23/25	59/64
19	CAGACTATCTGTGTCTG	3'UTR	3'SL1	0	11	619	17446	0	45	1774	43491	47.1	63	18/19	18/20	23/25	59/64
20	AGACTATCTGTGTCTGG	3'UTR	3'SL1	0	19	425	13464	0	26	1183	31593	47.1	70	19/20	18/20	23/25	60/65
21	GACTATCTGTGTCTGGG	3'UTR	3'SL1	0	19	312	10987	1	20	1255	26931	52.9	75	19/20	19/21	23/25	61/66
22	ACTATCTGTGTCTGGGT	3'UTR	3'SL1	0	20	337	11479	0	62	2687	34833	47.1	79	19/20	19/21	23/25	61/66
23	CTATCTGTGTCTGGGTG	3'UTR	3'SL1	0	19	715	15558	1	64	2433	51178	52.9	64	19/20	19/21	23/25	61/66
24	CCCCCCCTCTATTGAGG	3'UTR	3'SL2	0	1	17	2134	0	4	212	5463	64.7	89	0/84	20/31	14/25	34/140
25 (3)	CCCCCCTCTATTGAGGG	3'UTR	3'SL2	0	0	41	2241	0	5	186	4551	64.7	82	0/84	20/31	14/25	34/140
26	CCCCCTCTATTGAGGGG	3'UTR	3'SL2	0	2	44	3062	0	3	202	4722	64.7	83	0/84	20/31	14/25	34/140
27	CCCCTCTATTGAGGGGG	3'UTR	3'SL2	0	2	178	2992	0	6	195	4644	58.8	81	0/84	20/31	14/25	34/140
28	CCCTCTATTGAGGGGGA	3'UTR	3'SL2	0	0	235	4340	0	7	339	8105	58.8	88	0/84	20/31	14/25	34/140
29	CCTCTATTGAGGGGGAA	3'UTR	3'SL2	0	49	254	5184	0	7	413	8684	52.9	76	0/84	20/31	14/25	34/140
30	GAAGAGAGTTGCCAATT	3'UTR	3'SL4	0	3	350	9251	0	16	734	17325	41.1	72	0/87	0/31	24/25	24/143
31 (4)	AAGAGAGTTGCCAATTT	3'UTR	3'SL4	0	0	350	10380	0	24	842	19913	41.1	71	0/87	0/31	24/25	24/143
32	AGAGAGTTGCCAATTTC	3'UTR	3'SL4	0	4	343	9544	1	29	757	18542	41.1	85	0/87	0/31	24/25	24/143
33	GAGAGTTGCCAATTTCT	3'UTR	3'SL4	0	6	406	112327	0	18	791	19149	41.1	83	0/87	0/31	24/25	24/143
34	AGAGTTGCCAATTTCTC	3'UTR	3'SL4	0	11	530	13014	0	17	912	20604	41.1	80	0/87	0/31	24/25	24/143
35	GAGTTGCCAATTTCTCT	3'UTR	3'SL4	0	5	759	13284	0	27	1063	25278	41.1	84	0/87	0/31	25/25	25/143
36	AGTTGCCAATTTCTCTC	3'UTR	3'SL4	0	75	816	16376	0	18	1119	28101	41.1	81	0/87	0/31	25/25	25/143
37	GTTGCCAATTTCTCTCT	3'UTR	3'SL4	0	77	1168	19893	0	33	1471	31822	41.1	82	0/87	0/31	25/25	25/143
38	TTGCCAATTTCTCTCTT	3'UTR	3'SL4	0	134	2036	32834	1	66	2631	52962	35.3	68	0/87	0/31	25/25	25/143
39	TGCCAATTTCTCTCTTC	3'UTR	3'SL4	0	39	1837	34561	2	66	2572	53276	41.1	71	86/87	30/31	25/25	141/143
40	ACCATGTTCGGCCTTGT	3'UTR	3'SL6	0	9	176	5758	0	3	282	10804	52.9	89	1/90	0/31	7/25	8/146
41	CCATGTTCGGCCTTGTC	3'UTR	3'SL6	0	13	305	6770	0	5	258	8573	52.9	85	1/90	0/31	7/25	8/146
42	CATGTTCGGCCTTGTCA	3'UTR	3'SL6	0	13	298	7033	0	1	277	9458	52.9	74	1/90	0/31	7/25	8/146
43 (5)	ATGTTCGGCCTTGTCAT	3'UTR	3'SL6	0	0	294	5289	0	7	284	9764	47.1	72	1/90	0/31	7/25	8/146
44	TGTTCGGCCTTGTCATG	3'UTR	3'SL6	0	2	310	6606	0	2	305	15611	52.9	69	1/90	0/31	7/25	8/146
45 (6)	GTTCGGCCTTGTCATGG	3'UTR	3'SL6	0	0	170	5799	0	3	308	9244	58.8	73	0/91	0/31	7/25	7/147
46 (7)	TTCGGCCTTGTCATGGT	3'UTR	3'SL6	0	0	406	6797	0	4	433	10657	52.9	87	0/91	0/31	7/25	7/147
47 (8)	TCGGGATCGACCATTTT	3'UTR	3'SL7	0	0	156	2849	0	1	90	3092	47.1	79	85/96	25/31	19/25	129/152
48 (9)	CGGGATCGACCATTTTC	3'UTR	3'SL7	0	0	264	3096	0	1	54	2272	52.9	73	89/96	25/31	19/25	133/152
49	GGGATCGACCATTTTCC	3'UTR	3'SL7	0	19	368	5053	0	3	199	5478	52.9	80	89/96	25/31	19/25	133/152
50	GGATCGACCATTTTCCC	3'UTR	3'SL7	0	94	243	5331	0	4	232	6228	52.9	84	89/96	24/31	19/25	132/152
51	GATCGACCATTTTCCCG	3'UTR	3'SL7	0	94	143	3126	0	0	76	2762	52.9	76	89/96	24/31	19/25	132/152
52	GGGTGATGGTGGCTCAG	3'UTR	3'SL1	0	54	3342	36451	4	82	2965	429666	64.7	74	17/21	29/30	25/25	71/76
53	TGGGTGATGGTGGCTCA	3'UTR	3'SL1	0	1023	7237	43168	7	509	7159	60566	58.8	80	17/21	29/30	25/25	71/76
54	CTGGGTGATGGTGGCTC	3'UTR	3'SL1	0	1025	7634	48412	6	452	6495	56173	64.7	79	17/21	27/28	25/25	69/74
55	TCTGGGTGATGGTGGCT	3'UTR	3'SL1	0	63	3044	36094	0	109	2602	39614	58.8	77	17/21	27/28	24/25	68/74
56	GTCTGGGTGATGGTGGC	3'UTR	3'SL1	0	43	4360	40278	1	184	5195	46209	64.7	83	21/21	26/27	24/25	71/73
57	CGAGTCACTCATCACCT	3'UTR	3'SL1	0	0	195	8815	0	4	432	12925	52.9	70	11/14	16/19	7/15	34/48
58 (11)	CCGAGTCACTCATCACC	3'UTR	3'SL1	0	0	271	8392	0	10	367	11092	58.8	83	11/14	16/19	7/15	34/48
59	TCCGAGTCACTCATCAC	3'UTR	3'SL1	0	8	232	11498	0	8	395	13179	52.9	86	11/14	16/19	7/15	34/48
60	GCTGTTGCTATCCGAAG	5'UTR	5'SL1	0	0	271	6307	0	23	1182	28523	52.9	64	0/25	0/34	12/25	12/84
61 (12)	CTGTTGCTATCCGAAGC	5'UTR	5'SL1	0	0	191	6510	0	22	1129	26667	52.9	63	0/25	0/34	12/25	12/84
62	TGTTGCTATCCGAAGCA	5'UTR	5'SL1	0	0	82	6281	0	23	1263	32566	47.1	59	0/25	0/34	12/25	12/84
63 (13)	GTTGCTATCCGAAGCAA	5'UTR	5'SL1	0	0	30	4006	0	37	1646	39692	47.1	70	0/25	0/33	16/25	16/83
64	TTGCTATCCGAAGCAAA	5'UTR	5'SL1	0	0	251	5556	0	4	336	8564	41.1	65	0/24	2/33	13/25	15/82
65 (14)	TGCTATCCGAAGCAAAC	5'UTR	5'SL1	0	0	76	4697	0	2	314	8700	47.1	61	6/24	27/33	6/16	39/73
66	GCTATCCGAAGCAAACG	5'UTR	5'SL1	0	0	55	2494	0	3	293	10887	52.9	61	6/23	27/33	6/16	39/72
67 (15)	CTATCCGAAGCAAACGC	5'UTR	5'SL1	0	0	59	2568	0	3	185	6633	52.9	49	6/23	27/33	6/16	39/72
68	AGCTCTTGTTCTCCTAA	5'UTR	5'SL3	0	14	592	15202	0	2	307	10076	41.1	65	110/110	36/36	27/27	173/173
69	GCTCTTGTTCTCCTAAG	5'UTR	5'SL3	0	1	547	16677	0	2	240	8850	47.1	59	110/110	36/36	26/27	172/173
70	CTCTTGTTCTCCTAAGC	5'UTR	5'SL3	0	3	885	17973	0	2	61	2950	47.1	69	110/110	36/36	26/27	172/173
71	TCTTGTTCTCCTAAGCT	5'UTR	5'SL3	0	31	831	24789	0	4	261	11108	41.1	61	110/110	36/36	26/27	172/173

Table S2: Human genomic sequences containing one mismatch or gap relative to LNA gapmer target sequences. Mismatched nucleotides and gaps are indicated in blue.

LNA gapmer	Genome sequence containing one mismatch or gap [5'→3'] (GRCh38/hg38 (Dec, 2013))	Chromosome	Potential off-target gene	Mismatch or gap position in LNA gapmer
1	TCCTTGTCAGGCTATCT	1	*MACF1*	DNA
	TCCTTGTCAGACTATTT	2	No annotated gene	LNA
	TCCTTGTCAGACT-TCT	2	*SPATA2L*	DNA
	TCCTTGTCAGACTTTCT	3	*RBMS3*	DNA
	TCCTTGTCAGACTA-CT	3	*RAB7A*	LNA
	TCCTTGTAAGACTATCT	5	No annotated gene	DNA
	TCCTTGTCAGACTACCT	5	No annotated gene	LNA
	TCCTTGTCAGATTATCT	5	No annotated gene	DNA
	TCCTTCTCAGACTATCT	9	No annotated gene	DNA
	TCCTTGTCAGACTAT-T	11	No annotated gene	LNA
	TCCTTGTCAGACTCTCT	11	No annotated gene	DNA
	TCCTTGTCAGACT-TCT	17	No annotated gene	DNA
	TCCTTGTCAGACTA-CT	18	*COLEC12*	LNA
	TCCTTGTCAGAC-ATCT	X	*CHST7*	DNA
	TCC-TGTCAGACTATCT	X	No annotated gene	DNA
	TCCTTGTCAGACAATCT	X	No annotated gene	DNA
	TCCTTGTCAGACT-TCT	X	No annotated gene	DNA
6	GTTCAGCCTTGTCATGG	6	*MLIP*	DNA
	GTTCGGCCTTGTCATTG	22	No annotated gene	LNA
8	TCTGGATCGACCATTTT	15	*ARHGAP11B*	LNA
	TCTGGATCGACCATTTT	15	No annotated gene	LNA
	TCGGGATCGA-CATTTT	18	*TCF4*	DNA
9	CGGGATCTACCATTTTC	4	*RP11-665G4.1*	DNA
Ctrl 1	ACTCT-GTCAACCAAT	1	No annotated gene	DNA
	ACTCTC-TCAACCAAT	1	*ZNF670*	DNA
	ACTCTC-TCAACCAAT	2	No annotated gene	DNA
	ACTCTC-TCAACCAAT	3	No annotated gene	DNA
	ACTCTC-TCAACCAAT	4	No annotated gene	DNA
	ACTCT-GTCAACCAAT	4	No annotated gene	DNA
	ACTCTC-TCAACCAAT	4	No annotated gene	DNA
	ACTCTC-TCAACCAAT	4	*HSPA4L*	DNA
	ACTCTC-TCAACCAAT	5	*CDH12*	DNA
	ACTCTC-TCAACCAAT	5	No annotated gene	DNA
	ACTCTC-TCAACCAAT	5	No annotated gene	DNA
	ACTCTC-TCAACCAAT	5	No annotated gene	DNA
	ACTCTCGTCAACCTAT	7	No annotated gene	LNA
	ACTCTC-TCAACCAAT	7	*KIAA1549*	DNA
	ACTCTC-TCAACCAAT	8	*PSD3*	DNA
	ACTCTC-TCAACCAAT	8	No annotated gene	DNA
	ACTCTAGTCAACCAAT	9	*DAPK1*	DNA
	ACTCTC-TCAACCAAT	10	No annotated gene	DNA
	ACT-TCGTCAACCAAT	10	No annotated gene	DNA
	ACTCTCGTAAACCAAT	10	No annotated gene	DNA
	ACTCTC-TCAACCAAT	13	No annotated gene	DNA
	ACTCTC-TCAACCAAT	14	No annotated gene	DNA
	ACTCTC-TCAACCAAT	16	No annotated gene	DNA
	ACTCT-GTCAACCAAT	16	*KCTD19*	DNA
	ACTCTCCTCAACCAAT	17	No annotated gene	DNA
	ACTCTCGTCAACCATT	19	No annotated gene	LNA
	ACTCT-GTCAACCAAT	20	*TMC2*	DNA
	ACTCT-GTCAACCAAT	X	No annotated gene	DNA
	ACTCTCGTCACCCAAT	X	*TRMT2B*	DNA
	A-TCTCGTCAACCAAT	X	No annotated gene	LNA
	ACTCT-GTCAACCAAT	X	*TTTY14*	LNA
Ctrl 2	GTAACT-GTCGTAACA	3	*FHIT*	DNA

Table S3: Human pre-spliced RNA sequences containing one mismatch or gap relative to LNA gapmer target sequences.

Mismatched nucleotides and gaps are indicated in blue.

LNA gapmer	Human pre-spliced RNA sequence containing one mismatch or gap［5'→3'］ (RefSeq curated on GRCh38/hg38.p14, D3G 26.03 (Mar, 2026))	Potential off-target gene	RefSeq accession no.	Mismatch or gap region in LNA gapmer
1	AGATAGTCTGA-AAGGA	*RREB1*	NM_001003698.4	DNA
	AGATAGTCTGA-AAGGA	*RREB1*	NM_001003699.4	DNA
	AGATAGTCTGA-AAGGA	*RREB1*	NM_001003700.2	DNA
	AGATA-TCTGACAAGGA	*ROBO2*	NM_001128929.3	DNA
	AGATAGTCTGA-AAGGA	*RREB1*	NM_001168344.2	DNA
	AGATAGTCTAGACAAGGA	*MARK1*	NM_001286124.2	DNA
	AGATAGTCTAGACAAGGA	*MARK1*	NM_001286126.2	DNA
	AGATAGTCTAGACAAGGA	*MARK1*	NM_001286128.2	DNA
	AGATA-TCTGACAAGGA	*ROBO2*	NM_001378190.1	DNA
	AGATA-TCTGACAAGGA	*ROBO2*	NM_001378191.1	DNA
	AGATA-TCTGACAAGGA	*ROBO2*	NM_001378192.1	DNA
	AGATA-TCTGACAAGGA	*ROBO2*	NM_001378194.1	DNA
	AGATA-TCTGACAAGGA	*ROBO2*	NM_001378195.1	DNA
	AGATA-TCTGACAAGGA	*ROBO2*	NM_001378196.1	DNA
	AGATA-TCTGACAAGGA	*ROBO2*	NM_001378198.1	DNA
	AGATA-TCTGACAAGGA	*ROBO2*	NM_001378199.1	DNA
	AGATA-TCTGACAAGGA	*ROBO2*	NM_001378200.1	DNA
	AGATA-TCTGACAAGGA	*ROBO2*	NM_001378201.1	DNA
	AGATA-TCTGACAAGGA	*ROBO2*	NM_001378203.1	DNA
	AGATA-TCTGACAAGGA	*ROBO2*	NM_001394212.1	DNA
	AGATA-TCTGACAAGGA	*ROBO2*	NM_001394213.1	DNA
	AGATA-TCTGACAAGGA	*ROBO2*	NM_001394214.1	DNA
	AGATA-TCTGACAAGGA	*ROBO2*	NM_001395657.1	DNA
	AGATAGTCTAGACAAGGA	*MARK1*	NM_018650.5	DNA
	AG-TAGTCTGACAAGGA	*COLEC12*	NM_130386.3	LNA
	AGGTAGTCTGACAAGGA	*LINC02208*	NR_104610.1	LNA
6	CCATGACAAGGCC-AAC	*GRID2*	NM_001286838.1	DNA
	CCATGACAAGGCC-AAC	*GRID2*	NM_001510.4	DNA
	CCATGACAAGGCC-AAC	*GRID2*	NM_001440459.1	DNA
8	AAAAT-GTCGATCCCGA	*TCF4*	NM_001243226.3	DNA
	AAAAT-GTCGATCCCGA	*TCF4*	NM_001243227.2	DNA
	AAAAT-GTCGATCCCGA	*TCF4*	NM_001243228.2	DNA
	AAAAT-GTCGATCCCGA	*TCF4*	NM_001369567.1	DNA
	AAAAT-GTCGATCCCGA	*TCF4*	NM_001369571.1	DNA
	AAAAT-GTCGATCCCGA	*TCF4*	NM_001369576.1	DNA
	AAAAT-GTCGATCCCGA	*TCF4*	NM_001369577.1	DNA
	AAAAT-GTCGATCCCGA	*TCF4*	NM_001369582.1	DNA
	AAAAT-GTCGATCCCGA	*TCF4*	NM_001369584.1	DNA
	AAAAT-GTCGATCCCGA	*TCF4*	NM_001369586.1	DNA
9	GAAAATGGTAGATCCCG	*C1QTNF7-AS1*	NR_125911.1	DNA

Table S3. Continued

LNA gapmer	Human pre-spliced RNA sequence containing one mismatch or gap [5'→3'] (RefSeq curated on GRCh38/hg38.p14, D3G 26.03 (Mar, 2026))	Potential off-target gene	RefSeq accession no.	Mismatch or gap region in LNA gapmer
Ctrl 1	ATTGGTTGA-GAGAGT	*ZNF670-ZNF695*	NR_037894.2	DNA
	ATTGGTTGA-GAGAGT	*ZNF670*	NM_001204220.2	DNA
	ATTGGTTGA-GAGAGT	*ZNF670*	NM_033213.5	DNA
	ATTGGTTTGACGAGAGT	*GPC6*	NM_005708.5	DNA
	ATTGGTTGAC-AGAGT	*KCTD19*	NM_001100915.3	DNA
	ATTGGTTGAC-AGAGT	*LOC102724542*	NR_187691.1	DNA
	ATTGGTTGAC-AGAGT	*LOC102724542*	NR_187692.1	DNA
	ATTGGTTGAC-AGAGT	*LOC102724542*	NR_187693.1	DNA
	ATTGGTTGAC-AGAGT	*LOC102724542*	NR_187694.1	DNA
	ATTGGTTGAC-AGAGT	*LOC102724542*	NR_187695.1	DNA
	ATTGGTTGAC-AGAGT	*LOC102724542*	NR_187696.1	DNA
	ATTGGTTGAC-AGAGT	*LOC102724542*	NR_187697.1	DNA
	ATTGGTTGAC-AGAGT	*LOC102724542*	NR_187698.1	DNA
	ATTGGTTGAC-AGAGT	*LOC102724542*	NR_187699.1	DNA
	ATTGGTTGAC-AGAGT	*LOC102724542*	NR_187700.1	DNA
	ATTGGTTGAC-AGAGT	*LOC102724542*	NR_187701.1	DNA
	ATTGGTTGAC-AGAGT	*LOC102724542*	NR_187702.1	DNA
	ATTGGTTGAC-AGAGT	*LOC102724542*	NR_187703.1	DNA
	ATTGGTTGAC-AGAGT	*LOC102724542*	NR_187704.1	DNA
	ATTGGTTGAC-AGAGT	*SPATS2L*	NM_015535.3	DNA
	ATTGGTTGAC-AGAGT	*SPATS2L*	NM_001282735.1	DNA
	ATTGGTTGAC-AGAGT	*SPATS2L*	NM_001282743.1	DNA
	ATTGGTTGAC-AGAGT	*SPATS2L*	NM_001100422.1	DNA
	ATTGGTTGAC-AGAGT	*SPATS2L*	NM_001100424.1	DNA
	ATTGGTTGAC-AGAGT	*SPATS2L*	NM_001100423.2	DNA
	ATTGGTTGAC-AGAGT	*SPATS2L*	NM_001282744.2	DNA
	ATTGGTTGA-GAGAGT	*ROBO2*	NM_001128929.3	DNA
	ATTGGTTGA-GAGAGT	*ROBO2*	NM_001378190.1	DNA
	ATTGGTTGA-GAGAGT	*ROBO2*	NM_001378191.1	DNA
	ATTGGTTGA-GAGAGT	*ROBO2*	NM_001378195.1	DNA
	ATTGGTTGA-GAGAGT	*ROBO2*	NM_001378196.1	DNA
	ATTGGTTGA-GAGAGT	*ROBO2*	NM_001378200.1	DNA
	ATTGGTTGA-GAGAGT	*ROBO2*	NM_001378201.1	DNA
	ATTGGTTGA-GAGAGT	*ROBO2*	NM_001378203.1	DNA
	ATTGGTTGA-GAGAGT	*ROBO2*	NM_001378192.1	DNA
	ATTGGTTGA-GAGAGT	*ROBO2*	NM_001378194.1	DNA
	ATTGGTTGA-GAGAGT	*ROBO2*	NM_001378198.1	DNA
	ATTGGTTGA-GAGAGT	*ROBO2*	NM_001378199.1	DNA
	ATTGGTTGA-GAGAGT	*ROBO2*	NM_001394212.1	DNA
	ATTGGTTGA-GAGAGT	*ROBO2*	NM_001394213.1	DNA
	ATTGGTTGA-GAGAGT	*ROBO2*	NM_001394214.1	DNA
	ATTGGTTGA-GAGAGT	*ROBO2*	NM_001395657.1	DNA
	ATTGGTTGA-GAGAGT	*CDH12*	NM_001317227.2	DNA
	ATTGGTTGA-GAGAGT	*CDH12*	NM_001364107.2	DNA
	ATTGGTTGA-GAGAGT	*CDH12*	NM_001364108.2	DNA
	ATTGGTTGA-GAGAGT	*CDH12*	NM_001364104.2	DNA
	ATTGGTTGA-GAGAGT	*CDH12*	NM_001364105.2	DNA
	ATTGGTTGA-GAGAGT	*CDH12*	NM_001364106.2	DNA
	ATTGGTTGA-GAGAGT	*CDH12*	NM_004061.5	DNA
	ATTGGTTGA-GAGAGT	*CDH12*	NM_001317228.1	DNA
	ATTGGTTGA-GAGAGT	*GUSBP15*	NR_034021.1	DNA
	ATTGGTTGA-GAGAGT	*HECW1*	NM_001287059.2	DNA
	ATTGGTTGA-GAGAGT	*HECW1*	NM_015052.5	DNA
	ATTGGTTGA-GAGAGT	*MKLN1*	NM_001145354.2	DNA
	ATTGGTTGA-GAGAGT	*KIAA1549*	NM_001164665.2	DNA
	ATTGGTTGA-GAGAGT	*KIAA1549*	NM_020910.3	DNA
	ATTGGTTGA-GAGAGT	*PSD3*	NM_001412895.1	DNA
	ATTGGTTGA-GAGAGT	*PSD3*	NM_001412896.1	DNA
	ATTGGTTGA-GAGAGT	*PSD3*	NM_206909.3	DNA
	ATTGGTTGA-GAGAGT	*PSD3*	NM_001412891.1	DNA
	ATTGGTTGA-GAGAGT	*PSD3*	NM_001412890.1	DNA
	ATTGGTTGA-GAGAGT	*PSD3*	NM_001412889.1	DNA
	ATTGGTTGA-GAGAGT	*PSD3*	NM_001412888.1	DNA
	ATTGGTTGA-GAGAGT	*PSD3*	NM_001412885.1	DNA
	ATTGGTTGA-GAGAGT	*PSD3*	NM_001412886.1	DNA
	ATTGGTTGA-GAGAGT	*PSD3*	NM_001362819.2	DNA
	ATTGGTTGA-GAGAGT	*PSD3*	NM_001412883.1	DNA
	ATTGGTTGA-GAGAGT	*PSD3*	NM_001412870.1	DNA
	ATTGGTTGA-GAGAGT	*PSD3*	NM_001412871.1	DNA
	ATTGGTTGA-GAGAGT	*PSD3*	NM_001412872.1	DNA
	ATTGGTTGA-GAGAGT	*PSD3*	NM_001412873.1	DNA
	ATTGGTTGA-GAGAGT	*PSD3*	NM_001412874.1	DNA
	ATTGGTTGA-GAGAGT	*PSD3*	NM_001412875.1	DNA
	ATTGGTTGA-GAGAGT	*PSD3*	NM_001412876.1	DNA
	ATTGGTTGA-GAGAGT	*PSD3*	NM_001412882.1	DNA
	ATTGGTTGA-GAGAGT	*PSD3*	NM_015310.4	DNA
	ATTGGTTGA-GAGAGT	*PSD3*	NM_001412865.1	DNA
	ATTGGTTGA-GAGAGT	*PSD3*	NM_001412866.1	DNA
	ATTGGTTGA-GAGAGT	*PSD3*	NM_001412880.1	DNA
	ATTGGGTGACGAGAGT	*TRMT2B*	NM_001167970.2	DNA
	ATTGGGTGACGAGAGT	*TRMT2B*	NM_001167971.2	DNA
	ATTGGGTGACGAGAGT	*TRMT2B*	NM_001167972.2	DNA
	ATTGGGTGACGAGAGT	*TRMT2B*	NM_024917.6	DNA
	ATTGGTTGAC-AGAGT	*TTTY14*	NR_125736.1	DNA
	ATTGGTTGAC-AGAGT	*TTTY14*	NR_158640.1	DNA
	ATTGGTTGAC-AGAGT	*TTTY14*	NR_125733.1	DNA
	ATTGGTTGAC-AGAGT	*TTTY14*	NR_125734.1	DNA
	ATTGGTTGAC-AGAGT	*TTTY14*	NR_125735.1	DNA
	ATTGGTTGAC-AGAGT	*TTTY14*	NR_158641.1	DNA
	ATTGGTTGAC-AGAGT	*TTTY14*	NR_125737.2	DNA
	ATTGGTTGAC-AGAGT	*TTTY14*	NR_001543.4	DNA
Ctrl 2	TGTTACGATGAGTTAC	*PHEX*	NM_000444.6	DNA
	TGTTACGAC-AGTTAC	*FHIT*	NM_001166243.3	DNA
	TGTTACGATGAGTTAC	*PHEX*	NM_001282754.2	DNA
	TGTTACGAC-AGTTAC	*FHIT*	NM_001320899.2	DNA
	TGTTACGAC-AGTTAC	*FHIT*	NM_001320900.2	DNA
	TGTTACGAC-AGTTAC	*FHIT*	NM_001320901.2	DNA
	GTTACGAC-AGTTAC	*FHIT*	NM_001354589.2	DNA
	TGTTACGAC-AGTTAC	*FHIT*	NM_001354590.2	DNA
	TGTTACGAC-AGTTAC	*FHIT*	NM_002012.4	DNA
	TGTTACGAC-AGTTAC	*FHIT*	NR_135491.2	DNA

Table S4: Design of optimized LNA gapmer candidates.

The selected optimized LNA gapmers are highlighted in blue. The numbers and letters in parentheses indicate the optimized LNA gapmer numbers. The blue letters represent LNA bases. MM, mismatch.

ASO ID	ASO sequence[5'→3']	Length (mer)	Target region	Target secondary structure	No. of complementary sequences in Human RNA (RefSeq human RNA release 225 (Jul, 2024))				No. of complementary sequences in Hamster genome (MesAur1.0 (Mar, 2013))				GC(%)	Tm value(℃)	No. of TBEV strains with the perfect match to ASO sequences			
					0 MM or gap	1 MM or gap	2 MM or gap	3 MM or gap	0 MM or gap	1 MM or gap	2 MM or gap	3 MM or gap			European subtype	Siberian subtype	Far-eastern subtype	Total
12(1)	**TCC**TTGTCAGACTA**TCT**	17	3'UTR	3'SL1	0	0	435	15417	1	49	1278	32160	41.1	89	14/17	16/19	23/24	53/60
12-1	**TCCT**TGTCAGACTA**TCT**	17	3'UTR	3'SL1	0	0	435	15417	1	49	1278	32160	41.1	89	14/17	16/19	23/24	53/60
12-2	**TCC**TTGTCAGACT**ATCT**	17	3'UTR	3'SL1	0	0	435	15417	1	49	1278	32160	41.1	93	14/17	16/19	23/24	53/60
12-3	**TCCT**TGTCAGACT**ATCT**	17	3'UTR	3'SL1	0	0	435	15417	1	49	1278	32160	41.1	93	14/17	16/19	23/24	53/60
12-p3'-1	**TCC**TTGTCAGACTAT**CTG**	18	3'UTR	3'SL1	0	0	155	5064	1	9	436	12819	44.4	76	14/17	16/19	23/24	53/60
12-p3'-2	**TCCT**TGTCAGACTAT**CTG**	18	3'UTR	3'SL1	0	0	155	5064	1	9	436	12819	44.4	75	14/17	16/19	23/24	53/60
12-p3'-3 (1A)	**TCC**TTGTCAGACTA**TCTG**	18	3'UTR	3'SL1	0	0	155	5064	1	9	436	12819	44.4	84	14/17	16/19	23/24	53/60
12-p3'-4	**TCCT**TGTCAGACTA**TCTG**	18	3'UTR	3'SL1	0	0	155	5064	1	9	436	12819	44.4	84	14/17	16/19	23/24	53/60
12-p5'-1	**CTC**CTTGTCAGACTA**TCT**	18	3'UTR	3'SL1	0	0	159	4755	0	12	356	9126	44.4	82	14/17	16/19	23/24	53/60
12-p5'-2	**CTCC**TTGTCAGACTA**TCT**	18	3'UTR	3'SL1	0	0	159	4755	0	12	356	9126	44.4	89	14/17	16/19	23/24	53/60
12-p5'-3	**CTC**CTTGTCAGACT**ATCT**	18	3'UTR	3'SL1	0	0	159	4755	0	12	356	9126	44.4	84	14/17	16/19	23/24	53/60
12-p5'-4 (1B)	**CTCC**TTGTCAGACT**ATCT**	18	3'UTR	3'SL1	0	0	159	4755	0	12	356	9126	44.4	93	14/17	16/19	23/24	53/60
12-p5'+3'-1	**CTC**CTTGTCAGACTAT**CTG**	19	3'UTR	3'SL1	0	0	87	1672	0	4	96	2873	47.4	72	14/17	16/19	23/24	53/60
12-p5'+3'-2	**CTCC**TTGTCAGACTAT**CTG**	19	3'UTR	3'SL1	0	0	87	1672	0	4	96	2873	47.4	77	14/17	16/19	23/24	53/60
12-p5'+3'-3	**CTC**CTTGTCAGACTA**TCTG**	19	3'UTR	3'SL1	0	0	87	1672	0	4	96	2873	47.4	79	14/17	16/19	23/24	53/60
12-p5'+3'-4 (1C)	**CTCC**TTGTCAGACTA**TCTG**	19	3'UTR	3'SL1	0	0	87	1672	0	4	96	2873	47.4	84	14/17	16/19	23/24	53/60
12-m3'-1	**TCC**TTGTCAGACTA**TC**	16	3'UTR	3'SL1	0	21	1132	37179	2	72	2816	69498	43.8	87	14/17	16/19	23/24	53/60
12-m3'-2	**TCCT**TGTCAGACTA**TC**	16	3'UTR	3'SL1	0	21	1132	37179	2	72	2816	69498	43.8	86	14/17	16/19	23/24	53/60
12-m3'-3	**TCC**TTGTCAGACT**ATC**	16	3'UTR	3'SL1	0	21	1132	37179	2	72	2816	69498	43.8	91	14/17	16/19	23/24	53/60
12-m3'-4	**TCCT**TGTCAGACT**ATC**	16	3'UTR	3'SL1	0	21	1132	37179	2	72	2816	69498	43.8	90	14/17	16/19	23/24	53/60
12-m5'-1	**CC**TTGTCAGACTA**TCT**	16	3'UTR	3'SL1	0	4	1058	34103	1	77	2872	71287	43.8	77	14/17	17/20	23/24	54/61
12-m5'-2	**CCT**TGTCAGACTA**TCT**	16	3'UTR	3'SL1	0	4	1058	34103	1	77	2872	71287	43.8	77	14/17	17/20	23/24	54/61
12-m5'-3	**CC**TTGTCAGACT**ATCT**	16	3'UTR	3'SL1	0	4	1058	34103	1	77	2872	71287	43.8	81	14/17	17/20	23/24	54/61
12-m5'-4	**CCT**TGTCAGACT**ATCT**	16	3'UTR	3'SL1	0	4	1058	34103	1	77	2872	71287	43.8	80	14/17	17/20	23/24	54/61
45(6)	**GTT**CGGCCTTGTCA**TGG**	17	3'UTR	3'SL6	0	0	170	5747	0	3	308	9244	58.8	73	0/91	0/30	6/24	6/145
45-1	**GTTC**GGCCTTGTCA**TGG**	17	3'UTR	3'SL6	0	0	170	5747	0	3	308	9244	58.8	85	0/91	0/30	6/24	6/145
45-2	**GTT**CGGCCTTGTC**ATGG**	17	3'UTR	3'SL6	0	0	170	5747	0	3	308	9244	58.8	77	0/91	0/30	6/24	6/145
45-3	**GTTC**GGCCTTGTC**ATGG**	17	3'UTR	3'SL6	0	0	170	5747	0	3	308	9244	58.8	88	0/91	0/30	6/24	6/145
45-p3'-1	**GTT**CGGCCTTGTCAT**GGT**	18	3'UTR	3'SL6	0	0	46	1798	0	2	186	4322	55.5	84	0/91	0/30	6/24	6/145
45-p3'-2 (6A)	**GTTC**GGCCTTGTCAT**GGT**	18	3'UTR	3'SL6	0	0	46	1798	0	2	186	4322	55.5	94	0/91	0/30	6/24	6/145
45-p3'-3	**GTT**CGGCCTTGTCA**TGGT**	18	3'UTR	3'SL6	0	0	46	1798	0	2	186	4322	55.5	84	0/91	0/30	6/24	6/145
45-p3'-4	**GTTC**GGCCTTGTCA**TGGT**	18	3'UTR	3'SL6	0	0	46	1798	0	2	186	4322	55.5	94	0/91	0/30	6/24	6/145
45-p5'-1	**TGT**TCGGCCTTGTCA**TGG**	18	3'UTR	3'SL6	0	0	89	2213	0	0	94	3370	55.5	66	0/91	0/30	6/24	6/145
45-p5'-2	**TGTT**CGGCCTTGTCA**TGG**	18	3'UTR	3'SL6	0	0	89	2213	0	0	94	3370	55.5	72	0/91	0/30	6/24	6/145
45-p5'-3	**TGT**TCGGCCTTGTC**ATGG**	18	3'UTR	3'SL6	0	0	89	2213	0	0	94	3370	55.5	71	0/91	0/30	6/24	6/145
45-p5'-4 (6B)	**TGTT**CGGCCTTGTC**ATGG**	18	3'UTR	3'SL6	0	0	89	2213	0	0	94	3370	55.5	76	0/91	0/30	6/24	6/145
45-p5'+3'-1	**TGT**TCGGCCTTGTCAT**GGT**	19	3'UTR	3'SL6	0	0	2	613	0	0	40	1196	52.6	76	0/91	0/30	6/24	6/145
45-p5'+3'-2 (6C)	**TGTT**CGGCCTTGTCAT**GGT**	19	3'UTR	3'SL6	0	0	2	613	0	0	40	1196	52.6	82	0/91	0/30	6/24	6/145
45-p5'+3'-3	**TGT**TCGGCCTTGTCA**TGGT**	19	3'UTR	3'SL6	0	0	2	613	0	0	40	1196	52.6	76	0/91	0/30	6/24	6/145
45-p5'+3'-4	**TGTT**CGGCCTTGTCA**TGGT**	19	3'UTR	3'SL6	0	0	2	613	0	0	40	1196	52.6	82	0/91	0/30	6/24	6/145
45-m3'-1	**GTT**CGGCCTTGTCA**TG**	16	3'UTR	3'SL6	0	2	581	16791	0	7	796	30506	56.2	71	1/91	0/30	6/24	7/145
45-m3'-2	**GTTC**GGCCTTGTCA**TG**	16	3'UTR	3'SL6	0	2	581	16791	0	7	796	30506	56.2	84	1/91	0/30	6/24	7/145
45-m3'-3	**GTT**CGGCCTTGTC**ATG**	16	3'UTR	3'SL6	0	2	581	16791	0	7	796	30506	56.2	76	1/91	0/30	6/24	7/145
45-m3'-4	**GTTC**GGCCTTGTC**ATG**	16	3'UTR	3'SL6	0	2	581	16791	0	7	796	30506	56.2	86	1/91	0/30	6/24	7/145
45-m5'-1	**TT**CGGCCTTGTCA**TGG**	16	3'UTR	3'SL6	0	48	872	20693	0	10	1015	27685	56.2	64	0/91	0/30	6/24	6/145
45-m5'-2	**TTC**GGCCTTGTCA**TGG**	16	3'UTR	3'SL6	0	48	872	20693	0	10	1015	27685	56.2	77	0/91	0/30	6/24	6/145
45-m5'-3	**TT**CGGCCTTGTC**ATGG**	16	3'UTR	3'SL6	0	48	872	20693	0	10	1015	27685	56.2	69	0/91	0/30	6/24	6/145
45-m5'-4	**TTC**GGCCTTGTC**ATGG**	16	3'UTR	3'SL6	0	48	872	20693	0	10	1015	27685	56.2	81	0/91	0/30	6/24	6/145
